# Environmental drivers of annual population fluctuations in a trans-Saharan insect migrant

**DOI:** 10.1073/pnas.2102762118

**Published:** 2021-06-21

**Authors:** Gao Hu, Constanti Stefanescu, Tom H. Oliver, David B. Roy, Tom Brereton, Chris Van Swaay, Don R. Reynolds, Jason W. Chapman

**Affiliations:** ^a^Department of Entomology, College of Plant Protection, Nanjing Agricultural University, 210095 Nanjing, People’s Republic of China;; ^b^Natural Sciences Museum of Granollers, E08402 Granollers, Catalonia, Spain;; ^c^Centre de Recerca Ecològica i Aplicacions Forestals (CREAF), E08193 Bellaterra (Cerdanyola del Vallès), Catalonia, Spain;; ^d^School of Biological Sciences, University of Reading, Reading RG6 6AS, United Kingdom;; ^e^UK Centre for Ecology and Hydrology, Crowmarsh Gifford OX10 8BB, United Kingdom;; ^f^Butterfly Conservation, Wareham BH20 5QP, United Kingdom;; ^g^Dutch Butterfly Conservation, NL-6700 AM Wageningen, Netherlands;; ^h^Natural Resources Institute, University of Greenwich, Chatham ME4 4TB, United Kingdom;; ^i^Department of Computational and Analytical Sciences, Rothamsted Research, Harpenden AL5 2JQ, United Kingdom;; ^j^Centre for Ecology and Conservation, University of Exeter, Penryn TR10 9FE, United Kingdom;; ^k^Environment and Sustainability Institute, University of Exeter, Penryn TR10 9FE, United Kingdom

**Keywords:** insect migration, population dynamics, painted lady butterfly, Lepidoptera

## Abstract

Many latitudinal insect migrants including agricultural pests, disease vectors, and beneficial species show huge fluctuations in the year-to-year abundance of spring immigrants reaching temperate zones. It is widely believed that this variation is driven by climatic conditions in the winter-breeding regions, but evidence is lacking. We identified the environmental drivers of the annual population dynamics of a cosmopolitan migrant butterfly (the painted lady *Vanessa cardui*) using a combination of long-term monitoring and climate and atmospheric data within the western part of its Afro-Palearctic migratory range. Our population models show that a combination of high winter NDVI (normalized difference vegetation index) in the Savanna/Sahel of sub-Saharan Africa, high spring NDVI in the Maghreb of North Africa, and frequent favorably directed tailwinds during migration periods are the three most important drivers of the size of the immigration to western Europe, while our atmospheric trajectory simulations demonstrate regular opportunities for wind-borne trans-Saharan movements. The effects of sub-Saharan vegetative productivity and wind conditions confirm that painted lady populations on either side of the Sahara are linked by regular mass migrations, making this the longest annual insect migration circuit so far known. Our results provide a quantification of the environmental drivers of large annual population fluctuations of an insect migrant and hold much promise for predicting invasions of migrant insect pests, disease vectors, and beneficial species.

## Significance

The painted lady butterfly is an annual migrant to northern regions, but the size of the immigration varies by more than 100-fold in successive years. Unlike the monarch, the painted lady breeds year round, and it has long been suspected that plant-growing conditions in winter-breeding locations drive this high annual variability. However, the regions where caterpillars develop over winter remained unclear. Here, we show for the European summer population that winter plant greenness in the savanna of sub-Saharan Africa is the key driver of the size of the spring immigration. Our results show that painted ladies regularly cross the Sahara Desert and elucidate the climatic drivers of the annual population dynamics.

Insect migration occurs on an enormous scale (1), with billions of individuals undertaking multigenerational migrations between seasonally favorable climatic zones around the globe (2–6). These long-range migration cycles profoundly influence terrestrial ecosystems via the large-scale transfer of biomass, energy, and nutrients (4–8), the provision of ecosystem services (8–10), impacts on agricultural productivity (11), and spread of disease (5, 12); thus, it is imperative that we better understand insect movement patterns. Recently, there has been a step change in our knowledge of the year-round spatial distribution and migratory routes of a few well-studied species (13, 14), particularly the monarch butterfly (*Danaus plexippus*) (15, 16) and (to a lesser extent) the painted lady butterfly (*Vanessa cardui*) (17, 18). However, interannual population dynamics of such insect migrants remain poorly known. One of the characteristic features is the interannual variation in the abundance of the first wave of immigrants to reach the temperate zone, which can vary by several orders of magnitude between successive years (3, 11, 17, 19). It is generally believed that this variation is driven by the effect of winter climate on breeding success and survival in the tropical and subtropical winter-breeding regions, particularly when these regions are arid or semiarid (1, 3, 20).

**Fig. 1.**
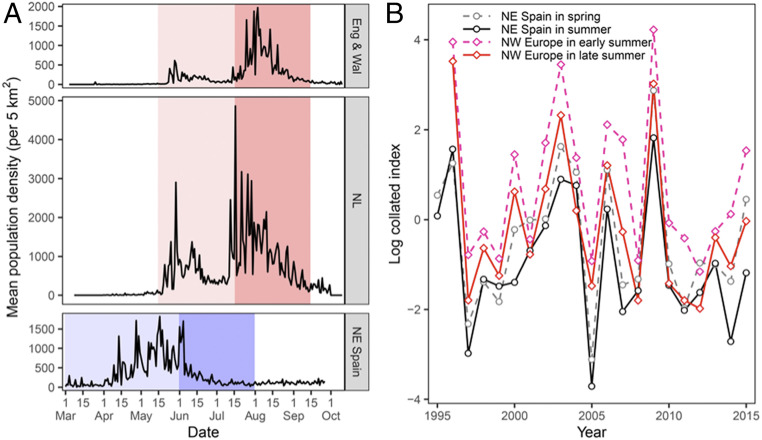
Painted lady population data in western Europe. (*A*) Phenology of painted ladies in Europe showing peaks that correspond to either migrants or local generations. In the Mediterranean region (NE Spain), the light-blue period corresponds to the spring immigration and the dark-blue period to the summer emergence of a locally bred generation. In NW Europe (NL: the Netherlands; Eng & Wal: England and Wales), the light-pink period corresponds to the early-summer immigration and the dark-pink period to the late-summer emergence of a locally bred generation. (*B*) Log-collated annual index (across all sites in each country) for NE Spain in spring (1 March to 30 May) and summer (1 June to 31 July) and for NW Europe in early summer (15 May to 15 July) and late summer (16 July to 30 September). Abundance indices are expressed on a log scale, with zero reflecting the average for that region and season across all years. See Fig. 4 for the factors explaining years of peak abundance (e.g., 1996, 2003, 2006, 2009, and 2015).

Here, we study the painted lady butterfly, a cosmopolitan, continuously breeding migrant that undertakes seasonally predictable, long-range movements between tropical/subtropical winter-breeding regions and temperate zone summer-breeding regions (17–20). We focus on the western portion of its Afro-Palearctic migration system (from the Gulf of Guinea to Fennoscandia) due to the unparalleled monitoring data available on the spring and summer generations in parts of this range (Butterfly Monitoring Scheme [BMS] data from western Europe; Fig. 1 *A* and *B*) and in order to quantify the environmental drivers of interannual variation in abundance. In this western section, winter breeding was traditionally considered to occur predominantly in the Maghreb region of northwestern (NW) Africa (21, 22). However, recent studies suggest that it can occur over a much larger latitudinal range, from the Gulf of Guinea coast to the north Mediterranean coast, with two-way movement across the Sahel and Sahara Desert linking European and sub-Saharan African populations (23–26). The colonization of Western Europe consists of a northward progression of successive generations throughout spring and summer. The European component starts when butterflies that had emerged in the Maghreb (Morocco, Algeria, and Tunisia) a few days previously (17, 27) arrive in the Mediterranean region during March and April and immediately produce the next generation there. What is unclear is just how important the winter generations produced south of the Sahara are in seeding or reinforcing the early-spring generation in the Maghreb. Subsequent late-spring and summer generations reach as far north as Fennoscandia, and then the autumn generation undertakes an extremely long migration [often high above the ground, utilizing fast tailwinds (17, 28)] back to NW Africa and sub-Saharan West Africa (17, 29). Here, the annual cycle, comprising six or more generations per year, resumes (17–20, 24).

**Fig. 2.**
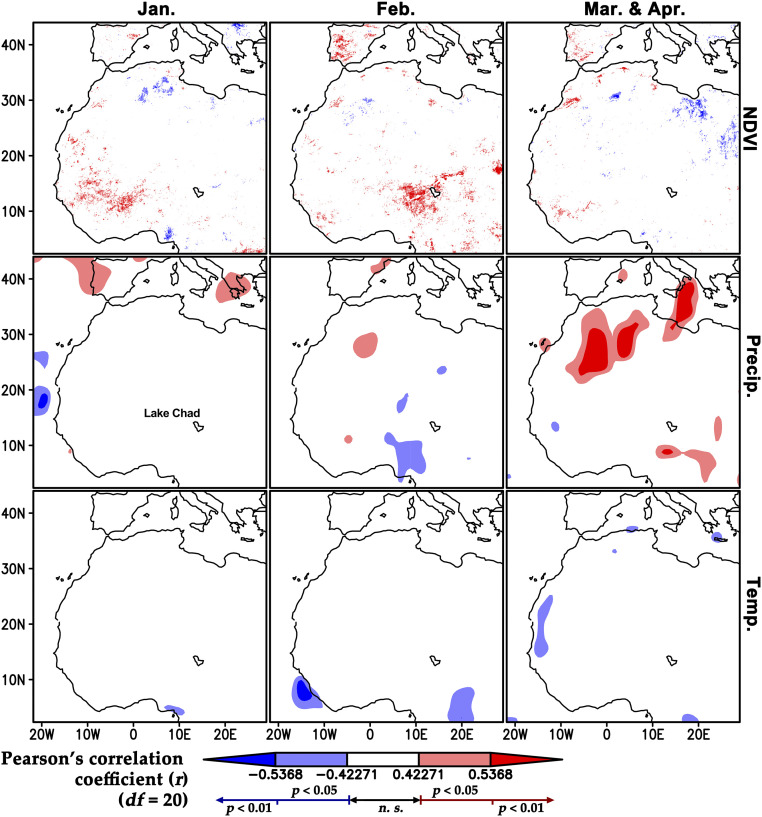
Correlations between spring painted lady counts in NE Spain with the NDVI, precipitation, and temperature. Red areas on the maps indicate regions that have positive significant correlations between the variable plotted and spring painted lady counts in NE Spain, while blue areas are negative correlations. See also Fig. 3 and *SI Appendix*, Fig. S2 for plots of painted lady spring numbers against the winter NDVI. These correlation plots were used to identify the ecoregions that were likely to be important (see delineation of these ecoregions in Fig. 3 and *SI Appendix*, Fig. S1) and to select the most important variables for the modeling (*SI Appendix*, Tables S1 and S2).

Extreme interannual variation in the abundance of the spring immigrants (and subsequent summer population) is a feature of painted lady population dynamics in both Europe (17, 19, 20, 30) and North America (29, 31, 32). Some painted ladies arrive in western Europe every spring; however, the pattern of abundance is one of irregular spectacular mass arrivals interspersed with years of much-reduced immigration (Fig. 1*B*). Here, we determine the key environmental conditions, and when/where they act during the migratory cycle, that drive this extreme annual variability in the European population dynamics each summer. In particular, we tackle the question of whether sub-Saharan, North African, and/or southern Iberian environmental conditions during the previous winter or spring are the primary drivers of the size of the spring and early-summer immigrations, initially to the Mediterranean and ultimately to northern Europe. To identify links between the generations monitored in Europe and the African breeding cycles both north and south of the Sahara, we use the following: 1) winter and spring environmental data (normalized difference vegetation index [NDVI], precipitation, temperature, and frequency of favorable tailwinds) covering the critical regions and periods (Fig. 2) in which large populations could potentially originate; 2) 21 y of BMS records from the Mediterranean (northeastern [NE] Spain) and NW Europe (the United Kingdom and the Netherlands); and 3) atmospheric trajectory simulations along the migratory route from sub-Saharan Africa to Europe.

## Results and Discussion

### Environmental Drivers of the Spring Migration to the Mediterranean.

**Fig. 3.**
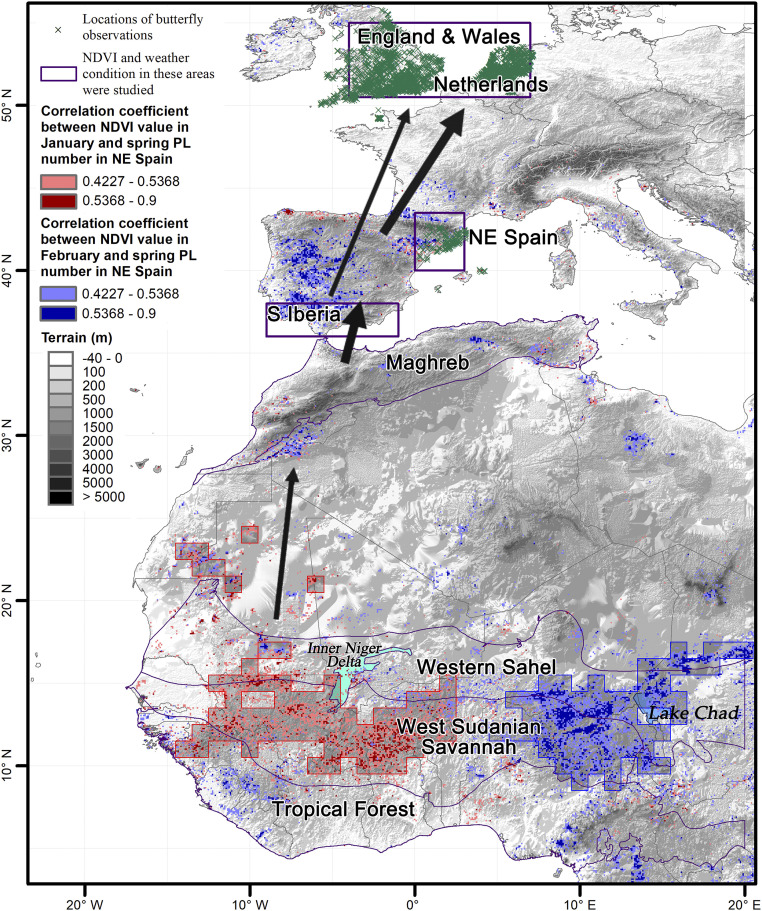
Migration arena of the painted lady. Regions implicated in driving population abundance of painted ladies in the western section of the Afro-Palearctic migratory range, according to the results of our preliminary correlation analyses. Regions labeled and outlined in purple are either potential source areas for winter breeding of painted ladies (“tropical forest,” “West Sudanian Savanna,” “Western Sahel,” “Maghreb,” and “Southern Iberia”) or regions where butterfly monitoring data were collected (green crosses in “NE Spain,” “Netherlands,” and “England & Wales”). Red and pink points superimposed on the map indicate areas with positive significant correlations between January NDVI values and spring painted lady counts in NE Spain, whereas dark- and light-blue points indicate areas with positive significant correlations with February NDVI values. The gray squares represent the “west kernel” (red) and “east kernel” (blue) used in our final models. See *SI Appendix*, Fig. S1 for a close-up view of the sub-Saharan region and the kernel areas.

The size of the annual spring immigration to NE Spain showed a high degree of interannual variation, with notable influxes in 1996, 2003, 2006, 2009, and 2015 (Fig. 1*B*); however, there was no evidence of a linear temporal trend in the size of the spring populations over the 21-y study period (*F* = 0.114, *P* = 0.739). Our first model selected the periods and ecoregions (i.e., large geographical units containing distinct assemblages of natural communities; Fig. 3 and *SI Appendix*, Fig. S1) that more strongly predicted the annual fluctuations in painted ladies reaching NE Spain in spring (model 1, *SI Appendix*, Tables S1 and S2). The full model included January to February NDVI from the three ecoregions south of the Sahara: Guinean tropical forest zone (hereafter “tropical forest”); West Sudanian Savanna (hereafter “Savanna”); and Western Sahel (hereafter “Sahel”); plus spring values from two ecoregions north of the Sahara: March to April NDVI from the Maghreb and April to May NDVI from South Iberia. Only January to February Savanna and March to April Maghreb NDVI were carried forward into our “minimum model,” and so all other ecoregions and periods were removed from further analyses. In this minimum model, Maghreb NDVI was significant (general linear mixed model; Maghreb NDVI_Mar–Apr_: *z* = 1.999, β coefficient = 0.512, SE = 0.256, *P* = 0.046; model 1, *SI Appendix*, Table S2); thus, as expected, more productive spring conditions in the Maghreb positively affected the number of migrant painted ladies reaching NE Spain.

Model 1 also indicated that NDVI averaged during January to February across the entire Savanna region (from the Atlantic coast to Lake Chad, see *SI Appendix*, Fig. S1) likely influenced the European population dynamics (Savanna NDVI_Jan–Feb_: *z* = 1.776, β coefficient = 0.444, SE = 0.250, *P* = 0.076; model 1, *SI Appendix*, Table S2). However, visual inspection of the outputs from the preliminary correlations (Fig. 2) showed a clear east/west temporal split: in the western part of the Savanna, the January NDVI was highly associated with spring arrivals, but in the eastern part of the region, it was the February NDVI that showed this relationship. This was confirmed by two models (2a and 2b, *SI Appendix*, Table S1) that selected NDVI values from only one of these two spatiotemporal combinations. Model 2a showed the importance of the January NDVI in the western subregion (NDVI_Jan_: *z* = 2.161, β coefficient = 0.528, SE = 0.244, *P* = 0.031), which was located mostly in the Savanna zone of southern Mali and Burkina Faso but with a small northward extension into the Sahel (southern Mauritania) and southern fringe of the Sahara (southeast Western Sahara and northeast Mauritania); we term this area the “west kernel” (see the gray squares with red outlines in Fig. 3 and *SI Appendix*, Fig. S1). Model 2b indicated a positive effect of the February NDVI in the eastern subregion centered on Lake Chad (NDVI_Feb_: *z* = 3.043, β coefficient = 0.690, SE = 0.227, *P* = 0.002), partly in the Savanna zone of northern Nigeria, partly in the Sahel of southeast Niger and southwest Chad, and extending marginally into the southern Sahara (in central Chad); we term this area the “east kernel” (see the gray squares with blue outlines in Fig. 3 and *SI Appendix*, Fig. S1). As these kernel areas straddle the two ecoregions, we refer to this key zone hereafter as the “Savanna/Sahel” region.

**Fig. 4.**
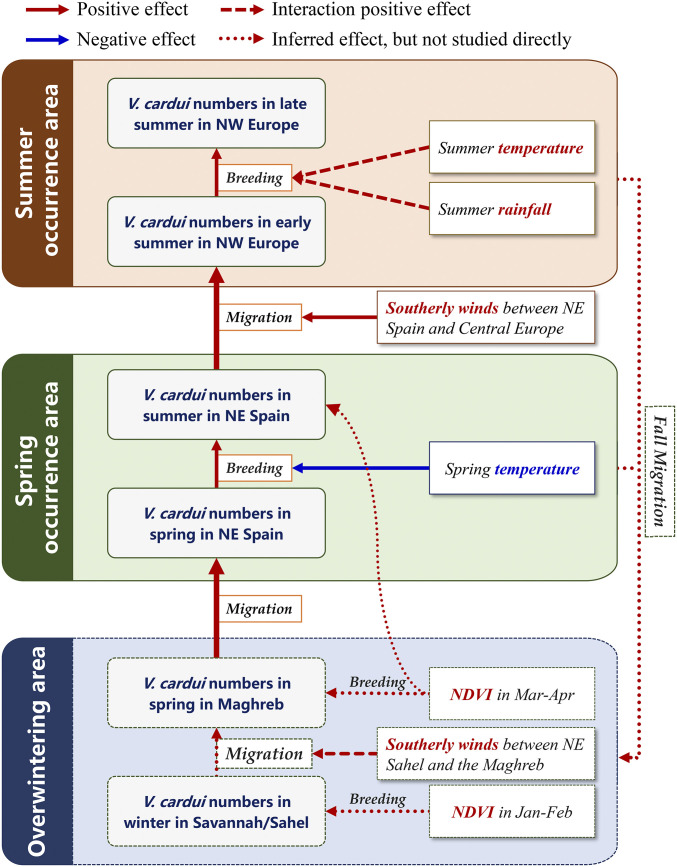
Schematic representation of some processes modeled in the analysis of the environmental drivers of painted lady migration intensity and population dynamics. The figure provides a summary of key results in understanding the northward migration of painted ladies from West Africa into western Europe. Environmental variables that positively affected population abundance are represented by solid red arrows for direct effects and dashed red arrows for an interaction effect, while variables that negatively affected population abundance are represented by blue arrows. Effects inferred but not directly studied (due to lack of population data in Africa) are indicated by dotted arrows. At the end of the summer breeding season, the European populations embark on a long fall migration back to the African winter-breeding grounds.

The discovery that Savanna/Sahel conditions are an important driver of European annual population fluctuations (Fig. 4) requires careful scrutiny, however, as it is unexpected for two reasons. First, the Savanna/Sahel is typically very dry during winter, and so it may seem surprising that this region can seemingly produce large numbers of painted ladies in certain years. Second, our results suggest that the migrants must be capable of successfully crossing the Sahara Desert against the seasonally prevailing winds, an enormous challenge for insects, which are comparatively short lived and weak flying with respect to migrant birds (33, 34). We discuss these two factors below.

### Geographical Origin of the Large Influxes to Europe.

The majority of migrant painted ladies arriving in southern Europe during spring (April to May) are generally assumed to be butterflies that emerged in the Maghreb a few days previously. This hypothesis is supported by three lines of evidence: 1) massive emergences in the Maghreb during some springs (21), 2) associations between the timing of the arrival in NE Spain and the occurrence of suitable high-altitude winds blowing from the Maghreb (27), and 3) the climatic suitability of the Maghreb for larval development during March and April (18). Our results showing that March and April Maghreb NDVI values are positively associated with the size of the spring immigration to NE Spain further reinforce this hypothesis. However, it is highly unlikely that painted ladies persist in large numbers in the Maghreb over winter, as this region is climatically less suitable for larval development from November to February (18); moreover, surveys indicate that populations largely disappear in midwinter (even following autumns with large arrivals from Europe) before rebounding again the following spring (22, 23). The reasons for the rapid population decline in autumn are likely to be that the combination of thermal limitation and the buildup of parasitoids in successive generations developing at the same location (35) promotes the emigration from the Maghreb of the butterflies emerging in late autumn. If the Maghreb is vacated over winter, what is the origin of the colonists arriving there each spring? Our model outputs show that winter conditions in the Savanna/Sahel region are important in driving the European population dynamics, and we consider that there are two non-mutually–exclusive hypotheses that may be responsible for this result, acting either singly or in concert.

The first hypothesis is that the abundance of painted ladies arriving in the Maghreb in spring is dependent on the productivity of a winter generation that develops in the Savanna/Sahel region in some years. This suggestion seems counterintuitive, as this extensive area has a dry season from winter to early spring (*SI Appendix*, Fig. S3*A*). Painted lady larvae develop on a wide range of ephemeral, fast-growing forbs that respond rapidly to water availability in arid regions (36, 37), and thus, in a typical year, winter breeding is probably largely confined to the more stable tropical forest region further south (18), with few of the progeny from this zone likely to be capable of reaching the Maghreb (see next section).

However, we suggest that conditions suitable for mass larval development in the Savanna/Sahel during the height of the dry season (December to February) occur in years with higher-than-average NDVI (*SI Appendix*, Fig. S2). High NDVI values are indicative of elevated plant productivity including the kind of arid-adapted annuals that larval painted ladies develop on, and this idea is supported by two additional lines of evidence. First, we found a highly significant positive relationship between NDVI and soil moisture in the west kernel during January (*r* = 0.736, *P* < 0.0001), indicating that a high NDVI is associated with greater water availability for winter plant growth and, as shown previously, with larger populations of herbivorous insects (38). Second, rainfall levels in the Savanna/Sahel kernel areas were notably higher than average in the autumns preceding a mass spring arrival in southern Europe (*SI Appendix*, Fig. S3*B*). This situation is known to facilitate development of large populations in very dry regions that are typically barren in midwinter: for example, millions of painted ladies emerged in Israel’s Negev Desert in winter 2015 to 2016 after developing on an abundance of quick-growing forbs (*Forsskaolea tenacissima* [Urticaceae] and *Malva parviflora* [Malvaceae]) that germinated in response to atypically high rainfall during the preceding autumn (37). Both of these larval host plants are present in the northern part of our kernel regions (39) and, given that the painted lady has one of the broadest diets known for any butterfly (36), it is very likely that other unrecorded host plants responding in a similar way will occur further south in these largely unmonitored areas. Importantly, all records of massive emergences come from very local, and sometimes highly isolated, spots of barely a few hectares (21, 24, 37), which means that egg-laying females have the ability to locate small potential breeding habitats that may be created by irregular episodes of intense autumn or winter rains (40).

The second hypothesis is that the high NDVI values reflect greater availability of nectar sources in the Savanna/Sahel, which could provide potential migrants with a critical opportunity to generate or replenish fuel loads, resulting in greater survival of migrants crossing the Sahara. A comparative situation occurs during the autumn migration of monarchs to Mexico, where floral availability along arid sections of the route is a determinant of migration-related mortality and thus, ultimately, one of the drivers of the size of the overwintering population (41–43). Thus, it is conceivable that nectar availability in the Savanna/Sahel is the primary factor driving the abundance of arrivals in the Maghreb and, ultimately, in Europe, although butterflies might actually originate in the tropical region. However, adult painted ladies typically emerge from the pupal stage with a full fat body (40), and comparative evidence from monarchs (44, 45) indicates that a full fat body contains enough fuel reserves for crossing the Sahara without the need to replenish fuel stores via nectar feeding (see next section). We therefore suggest that nectar availability is less likely to be the primary driver than the in situ production of large source populations, but that it may have an additional additive effect.

### Wind-borne Migration across the Sahara Desert.

**Fig. 5.**
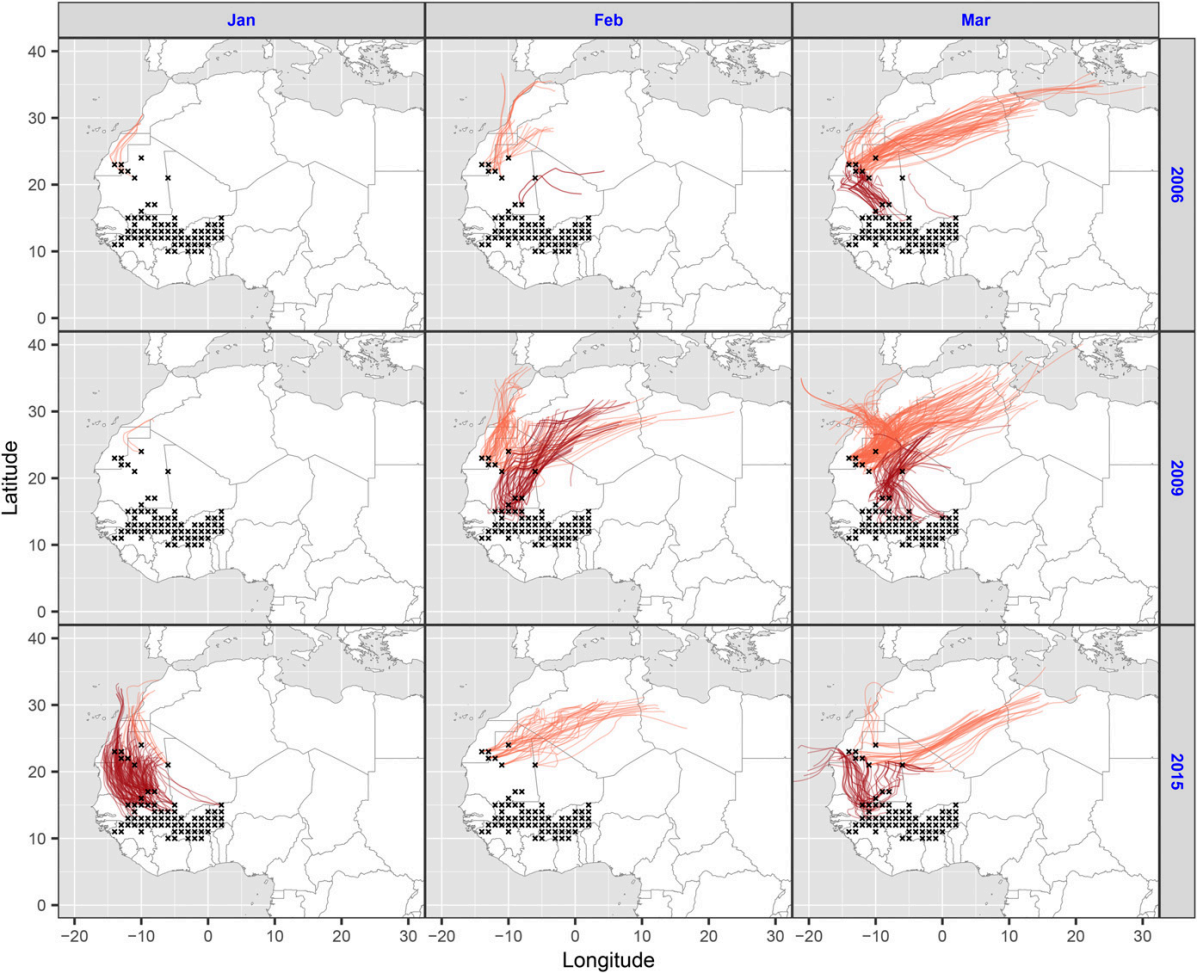
Simulated migration trajectories across the Sahara Desert. Forward migration trajectories for painted ladies from sub-Saharan West Africa during the spring of three recent years of mass immigration to NW Europe. The 4-d trajectories were started from each 1° × 1° cell (black crosses) in the west kernel of the Savanna/Sahel region: red lines show trajectories starting from the southern subregion of the kernel, while orange lines show trajectories from the northern subregion on the Sahel/Sahara border. Trajectory calculations involved daytime flight only for four successive days, including a self-powered flight vector of 6 m ⋅ s^−1^ toward the north at flight altitudes of 1,000, 1,500 and 2,000 m above ground level. In these plots, only “successful” trajectories are shown—these are defined as trajectories that cross the Sahara (reaching 28°N) from either subregion or reach the northern subregion from the southern part of the west kernel. The full 12-y dataset of successful trajectories from the west kernel is shown in *SI Appendix*, Fig. S4, while the complete sets of all trajectories (successful and unsuccessful) from the west and east kernels are shown in *SI Appendix*, Figs. S5 and S6, respectively.

The links we have identified between populations developing in the Savanna/Sahel and the Maghreb indicate that painted ladies must regularly engage in long-range wind-borne migration across the Sahara Desert in the late winter/early spring. We investigated the probability of such long-range movements with two complementary methods. First, we built models that included the speed of the southerly wind component (blowing along the proposed migratory route across the Sahara from the Savanna/Sahel kernel areas to the Maghreb) to see if this affected the size of the European immigration (models 3a and 3b, *SI Appendix*, Table S1). Southerly wind speed from the west kernel was not significant in isolation (southerly wind_Feb–Mar_: *z* = 1.093, β coefficient = 0.314, SE = 0.287, *P* = 0.274), but we did find a significant interaction whereby a high winter NDVI and faster southerly winds produced larger immigrations to NE Spain (southerly wind_Feb–Mar_ × NDVI_Jan_ interaction: *z* = 2.040, β coefficient = 0.588, SE = 0.288, *P* = 0.041; model 3a, *SI Appendix*, Table S2). However, southerly winds from the east kernel had no effect on the number of immigrants reaching NE Spain (model 3b, *SI Appendix*, Table S2). Second, we carried out migration trajectory simulations starting from the center of each of the 1° × 1° grid cells making up our west and east kernel areas of the Savanna/Sahel region (Fig. 5 and *SI Appendix*, Figs. S4–S6). Our 4-d trajectory analyses, starting on every day of January through March from the west kernel and February through April from the east kernel from 2004 to 2015, incorporated a series of assumptions about behavior, flight altitude, fuel reserves, and migration duration (see *Methods*). Given the uncertainty around some of these assumptions, the trajectories should be seen as indicative of what is feasible rather than as precise migration pathways.

Of the 221,076 trajectories from each of the 1° × 1° grid cells comprising the west kernel area, only 2,792 (1.3%) successfully crossed the Sahara and reached the Maghreb, but this included at least some crossings in 33 of the 36 monthly periods modeled (mean: 78 trajectories per month; range: 0 to 356; *SI Appendix*, Figs. S4 and S5 and Table S3). Most of these successful trajectories (86%) started from the northern section of the west kernel (total: 2,406; mean: 67 per month) with relatively few (14%) starting from the southern section (total: 386; mean: 11 per month; *SI Appendix*, Fig. S4 and Table S3). However, a greater proportion of trajectories from the southern section reached the northern section (total: 2,152; mean: 60 per month), and we consider it possible that, after refueling here, some of those butterflies may have migrated again to reach the Maghreb (*SI Appendix*, Fig. S4). The trajectory simulations thus demonstrate the feasibility of painted ladies crossing the Sahara from the west kernel of the Savanna/Sahel region and arriving in their next potential breeding ground (the Maghreb) in about 4 d, and the population modeling confirms the significance of these trans-Saharan movements for the European population dynamics. To illustrate the potential movements, we present trajectories from 3 y with large spring arrivals in Europe, confirming frequent opportunities for crossing in each year, especially in March 2006, February through March 2009, and January through March 2015 (Fig. 5). We also note that the well-studied migrations of the desert locust, *Schistocerca gregaria*, demonstrate that insect movements from the West African Sahel to the Maghreb do occur in winter; swarms move on spells of southerlies associated with disturbances that interrupt the prevailing northeasterlies (46).

By contrast, none of the trajectories from the east kernel (*n* = 189,036) reached the Maghreb region of NW Africa (*SI Appendix*, Fig. S6), supporting our modeling results indicating that southerly winds blowing from this region do not affect the European population dynamics. Thus, the mechanism by which the winter NDVI in the east kernel affects western European population dynamics remains unclear. Trajectories frequently did cross the Sahara along a northeast bearing, however, arriving in Egypt and the Levant, where another generation could be produced. The resulting adults may then expand westward, where they will meet and combine with the migrants moving northward along the western route, leading to the large arrivals observed in NE Spain and NW Europe in late spring and summer. This mechanism requires verification, but such a large-scale westward movement of painted ladies (and vagrant emperor dragonflies [*Anax ephippiger*]) out of the Middle East was observed in spring 2019 as they migrated through Cyprus (47).

### Environmental Drivers of Population Size in Europe.

As might be expected from the close correlation of their collated indices of abundance (Fig. 1*B*), the size of the summer generation in NE Spain was strongly related to the size of the immigration during the preceding spring (spring density: *z* = 4.469, β coefficient = 0.035, SE = 0.008, *P* < 0.0001; model 4, *SI Appendix*, Table S2), and just like the spring influx, there was no evidence of a linear temporal trend in the size of the summer population over the 21-y period (*F* = 1.05, *P* = 0.318). More surprisingly, North African environmental conditions also had a direct effect on the size of the summer generation (Maghreb NDVI_Mar–Apr_: *z* = 2.852, β coefficient = 0.719, SE = 0.252, *P* = 0.004; Fig. 4) in addition to the indirect effect via its impact on the size of the spring immigration. Akaike information criterion (AIC) values from model 4 indicated that three alternative minimum models were equally valid, and the effect of the spring Maghreb NDVI remained significant in all three (models 4a–c, *SI Appendix*, Table S2). However, local environmental conditions had a negative effect in two of them, with the model outputs indicating that summer generations were smaller following hotter springs (NE Spain temperature_Mar–May_: *z* = −2.195, β coefficient = −0.566, SE = 0.258, *P* = 0.028; model 4a) and also following springs with higher NDVI values (NE Spain NDVI_Mar–May_: *z* = −2.190, β coefficient = −0.635, SE = 0.290, *P* = 0.028; model 4b, *SI Appendix*, Table S2).

As expected (Fig. 1*B*), the size of the first generation reaching NW Europe was best explained by the size of the summer generation in NE Spain (Med summer collated index: *z* = 5.024, β coefficient = 0.830, SE = 0.165, *P* < 0.0001; model 5). Mirroring the pattern observed in NE Spain, the NW European early-summer influx also showed a high degree of interannual variation, with large immigrations in 1996, 2003, 2006, 2009, and 2015 (Fig. 1*B*); once again, there was no evidence of a linear temporal trend (*F* = 0.33, *P* = 0.572). North African conditions were no longer an important driver of immigration this far north (Maghreb NDVI_Mar–Apr_: *z* = 0.772, β coefficient = 0.178, SE = 0.230, *P* = 0.440; model 5, *SI Appendix*, Table S2), but southerly wind speed between NE Spain and NW Europe in early summer was positively associated with the size of the immigration (southerly wind_Jun_: *z* = 2.301, β coefficient = 0.403, SE = 0.175, *P* = 0.021; model 6, *SI Appendix*, Table S2 and Fig. 4). Following the same pattern (Fig. 1*B*), the size of the second generation in NW Europe was significantly associated with the size of the previous generation (NW Europe early-summer density: *z* = 24.48, β coefficient = 0.047, SE = 0.002, *P* < 0.0001; model 7, *SI Appendix*, Table S2 and Fig. 4), which once again showed no evidence of a linear trend with time (*F* = 1.33, *P* = 0.264). Local environmental conditions during larval development did not have a direct effect on the size of the late-summer generation (NW Europe temperature_Jun–Jul_: *z* = −0.095, β coefficient = −0.031, SE = 0.324, *P* = 0.924; NW Europe precipitation_Jun–Jul_: *z* = −1.595, β coefficient = −0.527, SE = 0.330, *P* = 0.111; model 7, *SI Appendix*, Table S2). However, there were significant interaction effects: late-summer abundance was highest following a large early-summer immigration and when both air temperature and rainfall during development were also high (density × temperature interaction term: *z* = 21.74, β coefficient = 0.036, SE = 0.002, *P* < 0.0001; density × precipitation interaction term: *z* = 6.369, β coefficient = 0.015, SE = 0.002, *P* < 0.0001; model 7, *SI Appendix*, Table S2 and Fig. 4).

The outputs of models 4 through 7, aimed at explaining population progression from southern to northern Europe, provide some important insights. First, it is clear that the spring NDVI in the Maghreb had an additional positive effect on the size of the summer generation locally produced in the Mediterranean region. We propose that this effect is mediated via production of an additional summer generation in mountainous areas in the Maghreb, an idea supported by observations (23) of freshly emerged painted ladies in the Rif and Middle Atlas Mountains in June. In years when spring production in the Maghreb is high, we would also expect a higher abundance of this mountain summer generation and, accordingly, increased migration to Europe. Second, as expected, our models showed a clear generational effect on the annual population growth: the size of the spring immigration to NE Spain had a direct effect on the size of the subsequent summer generation there (model 4); this determined the number of immigrants reaching NW Europe in early summer (model 5), and the size of that immigration strongly impacted the final (late-summer) generation produced in NW Europe (model 7). Third, the significant influence of southerly wind speeds in two stages of the European colonization, from the west kernel to the Maghreb (model 3a) and then again from NE Spain to NW Europe (model 6), reinforces earlier suggestions (17, 27, 28) that high-altitude wind-borne transport is an important component of painted lady migrations, contrary to the widely held perception that butterfly migration occurs predominantly close to the ground (1). Finally, notwithstanding the major effects of the size of the previous generation, local climate during larval development played an additional role in influencing the population growth of the European generations. In the Mediterranean, temperature had a negative effect on population growth (model 4a), presumably because hotter springs lead to wilting of the larval food plants (the similar negative effect of the high NDVI probably only reflects its positive correlation [*r* = 0.433] with temperature). By contrast, warm summers that are also not too dry produce the largest late-summer generation in NW Europe, as these conditions are presumably ideal for vigorous growth of the preferred larval hosts (model 7).

## Conclusions

Our modeling results strongly implicate winter conditions in sub-Saharan Africa as key determinants of the size of the spring immigration to southern Europe and, consequently, the subsequent generations that progressively colonize the whole of western Europe. The West Sudanian Savanna has a dry season in the winter and early spring, and thus the importance of this region for the European population dynamics may seem counterintuitive. The primary mechanism is presumably due to greater availability of larval host plants at this season in wetter years, allowing massive populations to build up. In addition, the greater availability of adult nectar sources throughout the Savanna/Sahel presumably also plays a crucial role in allowing migrants to replenish valuable fuel stores during migration. Despite the typically dry winter conditions prevalent in the Savanna/Sahel, these regions still support high populations of insectivorous birds through the winter (38, 48). Furthermore, mass aerial movements of insects frequently occur above this part of Africa as evidenced by aerial captures of huge numbers of migrating insects above the Sahel of Mali (5, 12) and lengthy stopovers by aerially foraging common swifts to feed up in these regions on their spring and autumn migrations (49). Painted ladies are thus just one of many insect species that breed in the moister regions further south before spring migration. Our results clearly demonstrate that only occasional years are capable of producing the large populations that cause the spectacular influxes of painted ladies to Europe, and so in most years the African breeding grounds act as a bottleneck (3, 18) constraining the size of the migrant population the following spring. Rainfall patterns in the Sahel are changing and are projected to continue to change but in complex ways: rainfall events are more intermittent but stronger and tend to occur later in the season and more often in the central region of the Sahel (50, 51). These changes will undoubtedly impact painted lady generations south of the Sahara and likely influence immigrations to Europe, but exactly how these changes will manifest is difficult to predict. Our discovery of the central importance of the sub-Saharan generations in determining the magnitude of the European populations shows that the annual migration of the painted lady, an annual round-trip of about 12,000 to 14,000 km between tropical West Africa and Scandinavia, is the longest regularly undertaken insect migration circuit that is currently known. When more is known about the migratory cycles of painted ladies in other parts of its global range, it will be most illuminating to compare them with our recent discoveries of the Afro-European migratory circuit.

## Methods

### Painted Lady Population Data.

We use population monitoring and climate and atmospheric data to identify the environmental drivers of annual population dynamics of migratory painted lady butterflies *V. cardui* (Lepidoptera, Nymphalidae) in the Western Palearctic. Here, we are particularly concerned with the western migratory route (from sub-Saharan West Africa through Morocco and Spain to NW Europe), as we have the most complete data from this subregion. We used BMS data on painted ladies to assess annual population dynamics over a 22-y period (1994 to 2015) in 1) the western Mediterranean region using data from the Catalan BMS in NE Spain and 2) the NW European region, involving data from the Dutch BMS and the UK BMS (but restricted to transects in England and Wales [Eng & Wal]). We restrict our analyses to these locations with long-term data, as we consider them largely representative of the situation in the western Mediterranean and NW European regions, not because we consider them to be particularly important destinations or specific locations of discrete generations. BMS data follow a standardized data collection to develop an index of annual population size (52). Data are produced at the level of individual transect routes of varying length (from 1 to 5 km in length, sampling a range of habitat types), with numbers of all butterfly species recorded using the “Pollard walk” methodology (52) throughout March to September in NE Spain and April to September in the Netherlands and Eng & Wal. The average number of transects per year (± SD) were 49 (±22) for the spring migration period and for the summer generation in NE Spain and 1,031 (±373) for the early-summer migration period and 1,013 (±363) for the late-summer emergence in Eng & Wal and the Netherlands. These data were used to produce the mean seasonal patterns of painted lady abundance in each region (Fig. 1*A*) and the collated annual index for each region (Fig. 1*B*) and was included in the population models (*SI Appendix*, Tables S1 and S2). The collated index is an index derived from a Poisson general linear model applied to the site counts, with effects for site and year and adjustments for overdispersion and serial correlation (53). The collated index is widely used as a measure of the yearly species’ abundance at the regional level in European BMS (54). In the present work, we use the collated index to measure the yearly abundance of *V. cardui* in the Mediterranean region (NE Spain) and NW Europe (UK and the Netherlands) (Fig. 1*B*).

The painted lady transect counts provide one data point per year to the analysis, which reflects multiple generations (with life cycles mostly completed in Africa). Typically, when analyzing butterfly population data, density in the previous year is included as a covariate to account for first-order temporal autocorrelation (55–57). We checked for any signs of autocorrelation in the data by testing data from all transects (with a minimum of more than 5 y data) for temporal autocorrelation of first-, second-, third-, and fourth-order effects. We compared autocorrelation coefficients with 95% CIs expected under a white-noise process and found no evidence of temporal autocorrelation at lags of up to 4 y (*P* < 0.01; see *SI Appendix* for the outputs of the tests and the 95% CIs).

We split painted lady count data into separate seasonal periods based on our knowledge of migration patterns and local phenology. In NE Spain, we defined a “spring” period from 1 March to 31 May, when counts can be largely attributed to immigration, and a “summer” period from 1 June to 31 July, when counts are primarily attributable to the emergence of a locally bred generation (Fig. 1*A*). We did not analyze Mediterranean counts from August onwards, as these are likely to be influenced by southward return migration from northern Europe (22). For NW European populations, we split analyses into an “early summer” period (15 May to 15 July, corresponding to immigration) and a “late summer” period (16 July to 30 September, corresponding to the emergence of a locally bred generation; Fig. 1*A*).

### Environmental Data.

We obtained environmental data from autumn through spring (September to April) for our analysis period (1994 to 2015) from key regions of NW Europe, southwestern Europe, NW Africa, and sub-Saharan West Africa that we hypothesized as being potentially important in driving the abundance of painted ladies in their European range (see *Hypothesis Testing*). We used the terrestrial “ecoregions” identified by Olson and colleagues (58) to separate our environmental data into manageable but “relatively large units of land containing a distinct assemblage of natural communities and species, with boundaries that approximate the original extent of natural communities prior to major land-use change” (58). The ecoregions that we used in our analyses (Fig. 2 and *SI Appendix*, Fig. S1) are defined as follows, from north to south:

• “Southern Iberia”: the southern portion of Spain and Portugal up to 38° N, with a Mediterranean climate and flora.
• “Maghreb”: the Canary Islands, most of Morocco, the northernmost fringe of Algeria, and the northern half of Tunisia, from the west coast at 13° W to 11° E, with a Mediterranean climate and flora.
• “Sahara Desert”: not included as a distinct region in our models, as we considered this an unlikely region for mass development of painted ladies (but see below).
• “Sahel”: the semiarid transition zone of grassland and *Acacia* shrub between the Sahara and the West Sudanian Savanna; we restrict our analyses to the western portion of the Sahel running from northern Senegal through southern Mauritania, central Mali, northern Burkina Faso, southwestern Niger, and western Chad (from the west coast at 17° W to 20° E), which we term the “Western Sahel” region.
• “West Sudanian Savanna”: the tropical savanna region immediately south of the Sahel, consisting of a mixture of grasslands and wooded areas, running from central Senegal and the Gambia through southern Mali, northern Ivory Coast, much of Burkina Faso, northern Togo, northern Benin, and northern Nigeria, from the west coast at 18° W to 15° E.
• “Guinean Forest–Savanna Mosaic” and “Guinean Moist Forests”: two latitudinally narrow ecoregions lying adjacent to each other from north to south, running along the Gulf of Guinea from Guinea-Bissau to Cameroon, from the west coast at 17° W to 20° E; they share a moist climate, and we merged these two ecoregions into a single “tropical forest” zone for the purposes of our models.

The environmental data used in our models to explain annual population variation were normalized NDVI values (assumed to provide a relative proxy for abundance of caterpillar host plants; see *Discussion*), temperature, precipitation, and wind speed toward the north (i.e., southerly, *v* component) and toward the east (i.e., westerly, *u* component) at the presumed flight height required for wind-assisted migration to the next breeding region. We selected 1,500 m above sea level as the altitude at which to analyze the effect of wind speed because, during spring, favorably directed winds typically occur only >1,000 m above sea level (59, 60). NDVI data were sourced from the Global Inventory Monitoring and Modeling System (NDVI3g.v1 third generation NDVI, available from (https://glam1.gsfc.nasa.gov/). These data were obtained in NetCDF format in a half-monthly, global, 1/12-degree lat-long gridded dataset. Precipitation data were obtained from the Climate Prediction Center Merged Analysis of Precipitation (CMAP) data, comprising monthly and 5-d global-gridded precipitation means since 1979 produced by the National Oceanic and Atmospheric Administration’s Earth System Research Laboratory (https://www.esrl.noaa.gov/). From the same source, we obtained monthly and daily global-gridded surface air temperature data. Geopotential height and *u*- (westerly) and *v*- (southerly) winds at 850 hPa (∼1,500 m above sea level and presumed to be suitable for wind-borne transport of painted ladies) were derived from National Center for Environmental Prediction (NCEP)/National Center for Atmospheric Research (NCAR) reanalysis data from 1948 to 2011. The CMAP and NCEP/NCAR data have a spatial resolution of 2.5°. Mean values across selected periods and regions were calculated using the *gimms* package (version 1.1.1) in R (version 3.5, https://www.r-project.org/).

Preliminary correlation analyses indicated that winter and spring NDVI in African ecoregions were stronger predictors of spring abundance of painted ladies in NE Spain than any metric of total precipitation and temperature (Fig. 2). They were therefore retained as the main environmental explanatory variable in subsequent models accounting for spring population size, and African precipitation and temperature data were not used further. These same analyses showed that there was a clear temporal east–west trend in sub-Saharan winter NDVI correlation values. Spring arrivals were strongly associated with a high January NDVI in the western part of the Savanna and adjacent areas in the Sahel and even the Sahara Desert but also with a high February NDVI in the equivalent eastern part. To further investigate this geographical pattern, we built finer models in which the West Sudanian ecoregion was replaced by two smaller regions that we call the “west kernel” and “east kernel” areas. These areas comprised all the 1° × 1° grid squares that contained >30 individual grid cells (each of 0.08° × 0.08° area) with a significant positive correlation between January and February NDVI and spring painted lady numbers in NE Spain. The west kernel area (with a high January NDVI correlation) lies in the central part of the West Sudanian Savanna (mainly Burkina Faso and southern Mali) and marginally extends northward into the Western Sahel and Sahara and southward into the Tropical Forest region (Guinea). The east kernel area (with a high February NDVI correlation) occupies the eastern parts of the West Sudanian Savanna (northern Nigeria) and Western Sahel (southern Niger) and marginally extends northeastward into the Sahara (western Chad) and southward into the tropical forest region (Nigeria and Cameroon) (Fig. 2 and *SI Appendix*, Fig. S1). Mean NDVI in the ecoregions plus the kernel areas during the periods selected (see *Hypothesis Testing*) were calculated via the *ave()* and *aave()* functions in the Grid Analysis and Display System software (GrADS, version 2.0, cola.gmu.edu/grads/). The population models are described in detail in the *Population Modeling* section and *SI Appendix*, Table S1.

### Hypothesis Testing.

Based on previous knowledge of the migration ecology of the painted lady (17, 21–27), we raised a series of predictions to be tested on the regions and periods that would influence population abundance in Europe.

#### Spring migration into the western Mediterranean.

Evidence indicates that source populations of migrants arriving in the Mediterranean region (NE Spain) in April through May (Fig. 1*A*) develop in early spring in semidesert and agricultural areas in the Maghreb (21, 25–27). In this region, painted lady larvae feed on ephemeral plants whose growth is heavily constrained by water availability; thus, we expect the abundance of spring immigrants to NE Spain to fluctuate in close synchrony with plant productivity (i.e., NDVI) in the Maghreb in the months previous to the arrival (March to April).

However, other regions may constitute alternative source areas. For example, a strategy involving shorter migratory movements could result in African migrants colonizing Southern Iberia and then the offspring of these migrants colonizing NE Spain in a second step. In that case, we would predict the NDVI in Southern Iberia in April to May (where plant productivity is also heavily constrained by water availability) to become the main explanatory variable. In addition, evidence of trans-Saharan migration (23–26) lead us additionally to suggest that North African populations could in turn be dependent on regular immigration from sub-Saharan winter-breeding populations. We therefore also tested the role of the winter (January to February) NDVI in the various sub-Saharan ecoregions, and windspeed of high-altitude southerly winds blowing across the Sahara, on abundance in NE Spain via an indirect effect on the size of the intervening generation in the Maghreb.

#### Breeding success in the Mediterranean and subsequent migration into NW Europe.

A local generation emerges in the Mediterranean in June to July as evidenced by the appearance of fresh butterflies at that time and the presence of immature stages in the previous 2 mo. We tested breeding success in the Mediterranean by correlating numbers of spring migrants with local summer butterflies. Most butterflies from this local generation leave the area upon emergence to colonize NW Europe (e.g., the United Kingdom and the Netherlands; Fig. 1*A*). We therefore expect a strong positive association between numbers in the Mediterranean in June to July and numbers recorded in NW Europe in summer, and also perhaps between the speed of high-altitude southerly winds and arrivals in NW Europe. We also tested the effect of the NDVI in spring in Southern Iberia because the whole Iberian Peninsula is likely to be the source area of migrants arriving in NW Europe, and increased plant productivity in this region will favor a larger population of migrants. Finally, we tested for effects of the Maghreb NDVI on NW European numbers, considering that long distance migration between these regions could still occur and should not be neglected from the models.

#### Breeding success in NW Europe.

In contrast to the complex pattern in the Mediterranean, the phenology of painted ladies in NW Europe is clearly bimodal, with a second peak in abundance in August corresponding to the emergence of a local generation (Fig. 1*A*). This second peak is consistently greater than the first, suggesting that breeding success in NW Europe may be critical for the whole migratory cycle. We built a model to explain breeding success considering that the most likely factors constraining population growth in NW Europe is unfavorable weather for larval development (cold temperatures and high rainfall).

### Population Modeling.

We fitted generalized linear mixed-effects models (GLMMs) to painted lady count data using the *lme4* package (61) in the program R (62). All explanatory variables were standardized before analysis to zero mean and unit variance. Correlations between explanatory variables were assessed before modeling, and all variables retained in the models had pairwise Pearson’s correlation coefficients <0.7. Stepwise deletion was used to remove nonsignificant terms from models to produce a minimum adequate model in each case. All GLMMs were fitted to count data with a specified Poisson error structure. As additional control variables, transect length (m) was included as a fixed-effect variable along with the number of days that the transect was recorded that year. To account for repeated sampling at each monitoring site and within different years, we included *Site_ID* and *Year* (categorical) as random intercepts.

### Migration Trajectories.

Because our models indicated that the NDVI in the west and east kernel areas were especially important in driving the European populations dynamics, we assessed the capability of painted ladies to cross the Sahel and Sahara using a new numerical trajectory model that takes account of self-powered flight vectors [as these are known to substantially alter trajectory pathways (3, 28)]. This trajectory model has been used to accurately predict migration pathways of other insect migrants (63, 64).

Painted ladies are known to use a combination of migratory flight close to the ground (within their flight boundary layer [FBL], where they can make ground into unfavorable headwinds) and also at heights of several hundreds to >1,000 m above the ground to take advantage of favorable tailwinds (17, 27, 28). It seems improbable that self-powered FBL migration alone, without any form of tailwind assistance, would allow butterflies to cross the ∼2,500 km north–south expanse of the Sahel/Sahara. During spring migration, wind directions from ground level up to ∼1,000 m are generally unfavorable for a northward crossing (59, 60). This means that butterflies attempting this crossing must either 1) fly into a headwind if they stay within their FBL close to the ground or 2) travel high above the ground (>1,000 m above ground level, where they are more likely to receive tailwind assistance). Some simple calculations show it is very unlikely that ground-level migrations into a headwind would allow successful crossing of the Sahel/Sahara. The self-powered flight speed of painted ladies during migratory flight is likely to be about 6 m/s (21.6 km/h). If flying into even a light headwind of 2 m/s (7.2 km/h), maximum movement speeds will be 4 m/s (14.4 km/h). At this speed, it would take 14.5 d of 12-h nonstop flight (or 7.2 d of 24-h nonstop flight) to cross this vast region within their FBL—and this would have to be achieved largely in the absence of suitable nectar sources. We therefore think it is far more likely that high-altitude migration utilizing favorably directed tailwinds is used to cross this region.

We calculated potential forward migration trajectories during late winter and early spring from starting points within the west and east kernels to assess the likelihood that painted ladies can cross the Sahel and Sahara Desert and colonize the Maghreb region. The program for calculating trajectories was designed in Fortran (64) and run under CentOS 7.4 on a server platform (IBM system ×3500 M4). The Weather Research and Forecasting (WRF) model (version 3.8, https://www.mmm.ucar.edu/wrf-model-general) was used to produce a high-resolution atmospheric background for the trajectory calculations. The WRF is an advanced meso-scale numerical weather prediction system (65) (https://www.mmm.ucar.edu/weather-research-and-forecasting-model). National Centers for Environmental Prediction (NCEP) Final Analysis (FNL) data were used as the meteorological data for the model input. FNL is a six-hourly, global, 1-degree grid meteorological dataset. The model forecast time is 72 h with data outputs at 1 h intervals for horizontal and vertical wind speeds, temperature, and precipitation.

The flight behavior of painted ladies was included in the trajectory simulation by making the following assumptions:

1) Day-flying butterflies perform “multistop” migration, in which butterflies fly for much of the day before terminating migratory flight before sunset and then take off again the following morning. Such stop–start migration is typical of songbirds that cross the Sahara at a similar time (59, 60), albeit the songbirds migrate at night rather than during the day. We modeled flight trajectories from 07:00 to 18:00 local time (maximum of 11 h of flight per day).
2) Wind directions over the Sahara only become favorably directed for northward transport at heights of 1,000 to 3,000 m above mean sea level (amsl) (59, 60). Painted ladies are known to make use of high-altitude, favorable tailwinds (17, 27, 28) and have been directly observed (via telescope observations) flying at heights up to at least 2,000 m above the Sahara in Mauritania (17). Thus, to ensure we would capture the most likely flight height, we started trajectories from three different altitudes: 1,000, 1,500 and 2,000 m amsl.
3) We assumed that painted ladies are unable to maintain migratory flight activity whenever the air temperature at flight altitude falls below 10.0 °C, so trajectories were terminated on any day/height combination that dropped below this temperature. However, such cool temperature conditions rarely occurred.
4) Painted ladies were assumed to fly for four consecutive days (of 11 h of flight per day, thus a maximum total of 44 h) whenever temperature conditions at flight height were suitable. Monarch butterflies of the autumn migratory generation typically eclose from the pupal stage with a completely full fat body, indicating that they are ready to migrate before feeding on nectar (44). In addition, they are capable of replenishing their fat body from just a few hours of nectar feeding (44), indicating that frequent short stops for refueling during periods of migratory flight will allow them to keep their fat bodies topped up. A completely full fat body in a monarch can power a maximum of about 40 h of nonstop flight (45). There is no similar published data for the painted lady, so, in the absence of quantitative data, we have assumed that painted ladies will be similar to monarchs in terms of fuel loads and duration of flight powered by a full fat body (i.e., we assume that a newly eclosed adult painted lady leaving the kernel areas will have a full fat body allowing ∼40 h of flight without refueling) and that the fat body can be replenished at any suitable nectar sources encountered on the journey by just a few hours of feeding every 2 or 3 d. As well as any nectar sources encountered in the savanna and Sahel regions, refueling within the Sahara cannot be discounted, as the effect of the sparse winter rains may be enhanced by runoff in and around highlands such as the Ahaggar and the Adrar des lforas (46). To summarize, this means that our modeled trajectories lasting 4 d (44 h) of nonstop flight are approximately what can be powered by a single fat-body load, so refueling may not be necessary. We do not know enough about the flight strategies of painted ladies to judge what is the most realistic method to model, so we use a simple trajectory method (based on four successive days of continuous daytime flight interspersed with nighttime resting) to provide some insight into the feasibility of crossing the Sahara. It is important to note, therefore, that these trajectories are indicative and are not meant to represent realistic migratory pathways and timeframes.
5) Painted ladies fly with a self-powered airspeed of 6.0 m/s. The self-powered airspeed of a range of actively migrating butterflies of similar body size has been reported from field observations as follows: painted ladies at 4.9 to 7.1 m/s (average 6.0 m/s; ref. 19); *Catopsilia florella* (the African migrant or African emigrant butterfly, a species similar in size to the painted lady) at 5.4 to 7.1 m/s (average 6.25 m/s; ref. 66); and a suite of neotropical nymphalid (*Marpesia* spp. and *Historis acheronta*) and pierid butterflies (*Aphrissa* spp. and *Phoebis argante*, some of which are smaller than painted ladies) at 3.7 to 6.6 m/s (average 5.2 m/s; ref. 67). Thus, we assumed that a self-powered airspeed of 6.0 m/s, the mean value of the ranges above, would be typical of migrating painted ladies, and we added this value to the wind speed in the trajectory modeling.
6) Painted ladies will show a preferred flight heading toward the north in the winter/spring. Painted ladies are known to use a sun compass to select seasonally beneficial migration headings (68), and radar observations from Europe have previously shown that painted ladies can head toward the north during spring migrations (17, 28). Thus, the 6 m/s self-powered flight vector was directed toward the north irrespective of wind conditions in our trajectory modeling.

We investigated the possibility that painted ladies may cross the Sahara from the west and east kernel areas identified in our analyses as being of importance in the population dynamics of European populations (the gray squares in Fig. 2 and *SI Appendix*, Fig. S1). To model these potential migration routes during 2004 to 2015, trajectories were started from all potential departure points at every 1° × 1° grid cell in the west kernel area for every day of January, February, and March (Fig. 5 and *SI Appendix*, Figs. S4 and S5) and from the east kernel area for every day of February, March, and April (*SI Appendix*, Fig. S6).

## Supplementary Material

Supplementary File

Supplementary File

## Data Availability

Excel spreadsheets of climate and population data have been deposited in Dryad, https://doi.org/10.5061/dryad.j6q573ndv (69).
