# Supplementary material for: Environmental drivers of annual population fluctuations in a trans-Saharan insect migrant
Source: Proc Natl Acad Sci U S A. 2021 Jun 21;118(26):e2102762118. doi: 10.1073/pnas.2102762118 (PMC8256005; doi:10.1073/pnas.2102762118)
Supplement: Supplementary File [file pnas.2102762118.sapp.pdf]

Fig. S1. Close-up of the kernel areas in the Savannah/Sahel region of sub-Saharan Africa. The "kernels" used in our models were defined as all the 1° x 1° grid squares in our African study region which contained >30 individual grid cells (each of 0.08° x 0.08° area) which had a significant positive correlation between: (i) **January** NDVI values and spring painted lady numbers in NE Spain (grey squares with red outlines); or (ii) **February** NDVI values and spring painted lady numbers in NE Spain (grey squares with blue outlines). This process led to our selecting two kernels, one in the west for January data, and one in the east for February data. The west kernel lies mostly within the western portion of the West Sudanian Savannah ecoregion (mostly within southern Mali and Burkina Faso), but it extends northwards into limited areas of the Western Sahel and with an isolated area in the southern fringe of the Sahara (mostly on the border of Western Sahara and Mauritania); it also extends marginally into the northernmost fringe of the western tropical forest region in Guinea. The east kernel is centred on the Lake Chad region, and lies mostly in the eastern portion of the West Sudanian Savannah ecoregion (mostly in northern Nigeria) and the Western Sahel (mostly southeast Niger and southwest Chad), but extending marginally into the southern fringe of the Sahara in central Chad.

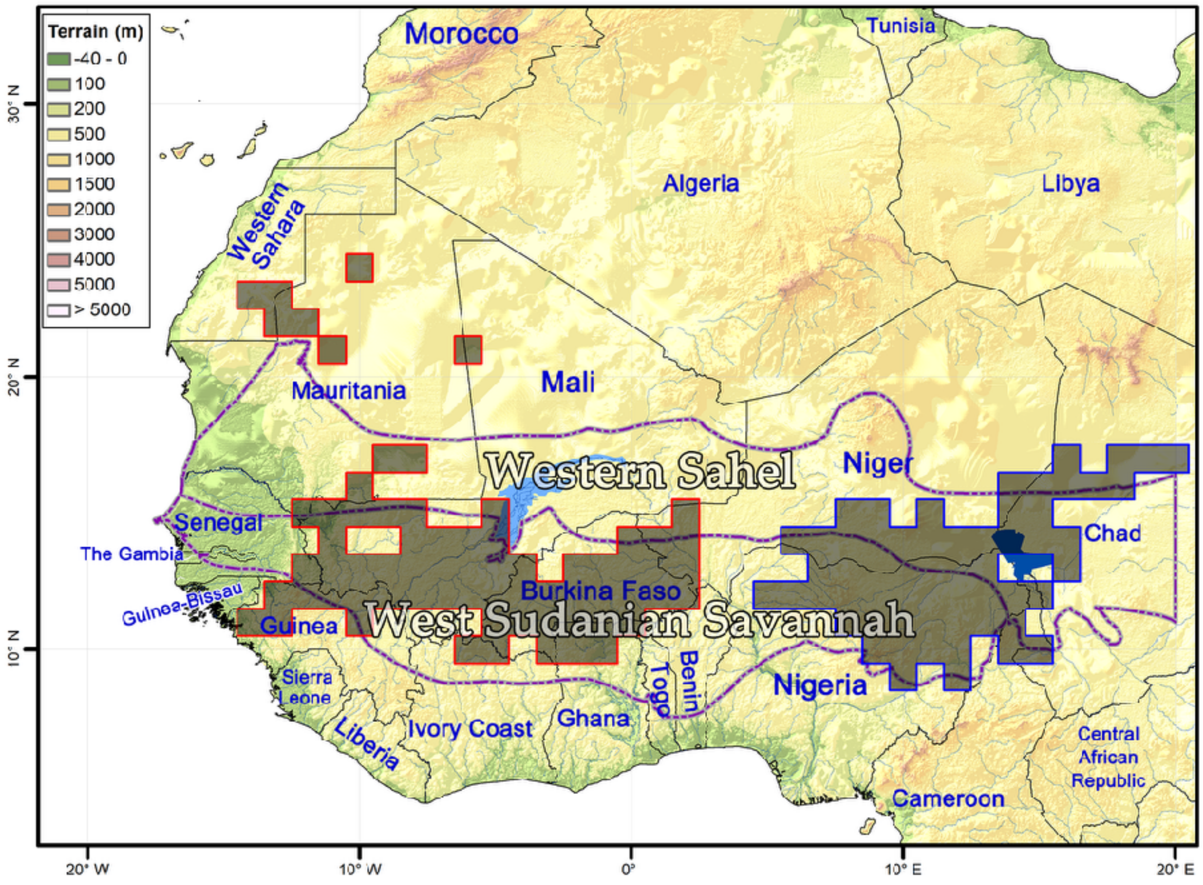

Fig. S2. Relationships between African NDVI and painted lady abundance in Europe. The mean log abundance of painted ladies reaching NE Spain in each spring plotted against January NDVI in the west kernel, February NDVI in the east kernel, and Mar–Apr NDVI in the Maghreb. In each case there is a significant positive relationship (linear regressions; west kernel $NDVI_{Jan}$: n=815, r^2=0.073, P<0.0001; east kernel $NDVI_{Feb}$: n=815, r^2=0.163, P<0.0001; Maghreb $NDVI_{Mar–Apr}$: n=815, r^2=0.148, P<0.0001).

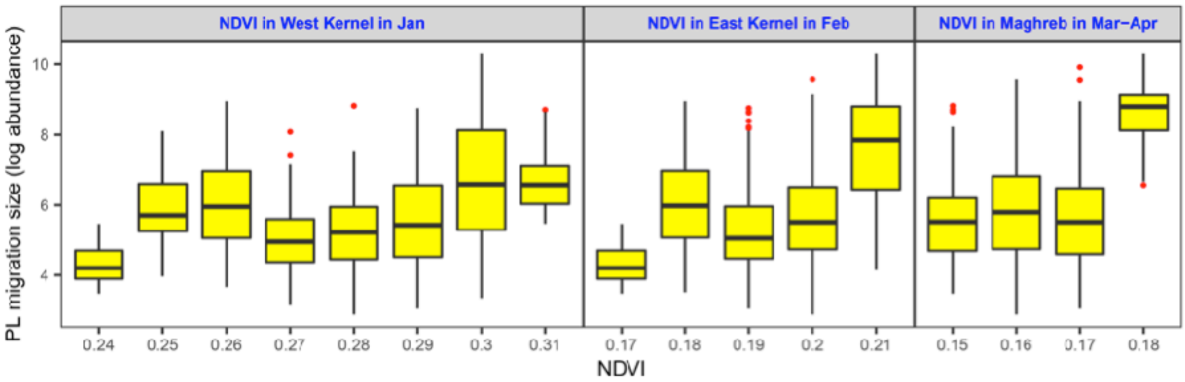

Fig. S3. Annual pattern of rainfall in the West Sudanian Savannah. (*A*) Mean daily rainfall in each month in the west kernel (largely in the West Sudanian Savannah) during 1993–2015. (*B*) Annual rainfall values during Oct–Dec in the west kernel, and Oct–Jan in the east kernel, compared to the overall mean (dashed line), with each of the autumn/early-winter periods preceding a mass invasion year indicated in red (e.g. 1995 shown in red as it precedes the 1996 mass arrival) while autumns preceding non-mass arrival years are shown in blue. Autumns preceding mass arrival years have higher than average rainfall levels in at least one month in either (or both) of the west and east kernels.

(***A***)

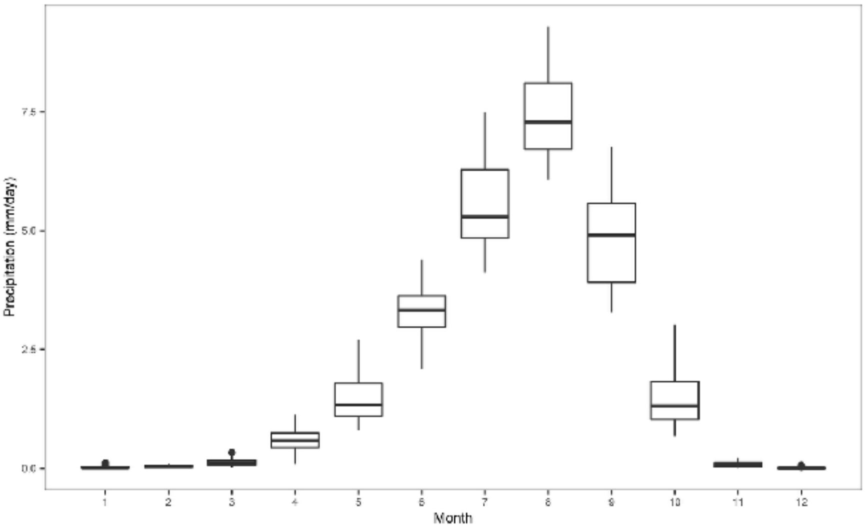

(***B***)

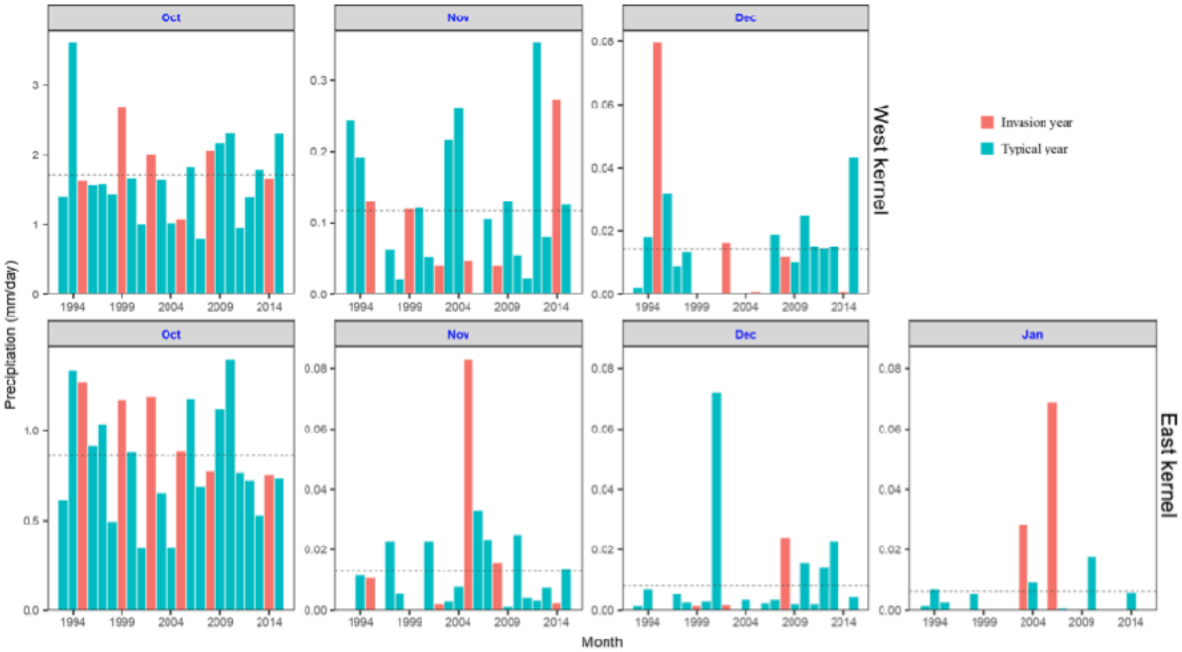

Fig. S4. Migration trajectories across the Sahara Desert. Four-day trajectories from the west kernel: red lines show trajectories from the southern sub-region of the kernel, while orange lines show trajectories from the northern sub-region. In these plots, only “successful” trajectories are shown – those that cross the Sahara from either sub-region, or reach the northern sub-region from the southern part of the west kernel.

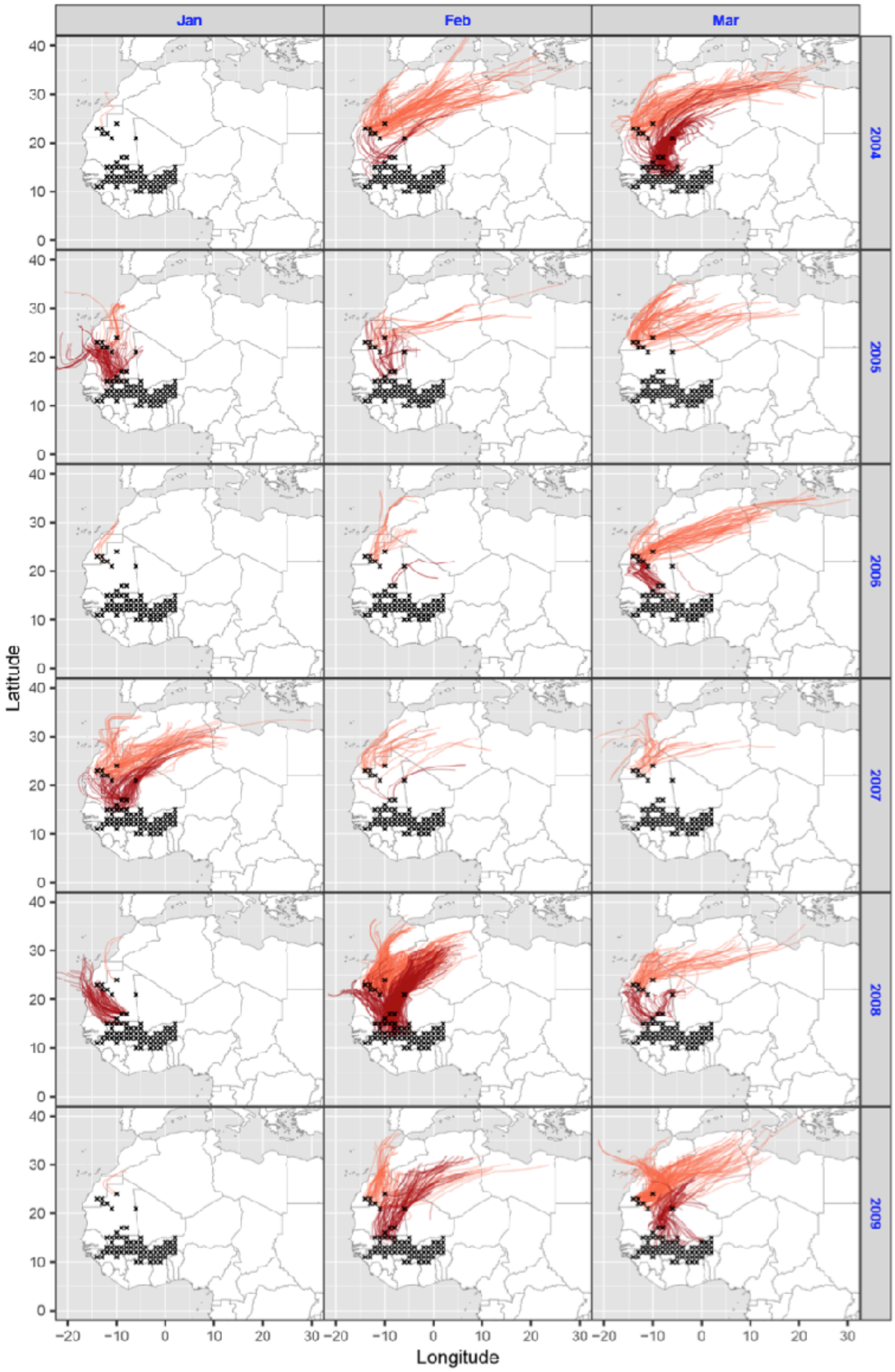

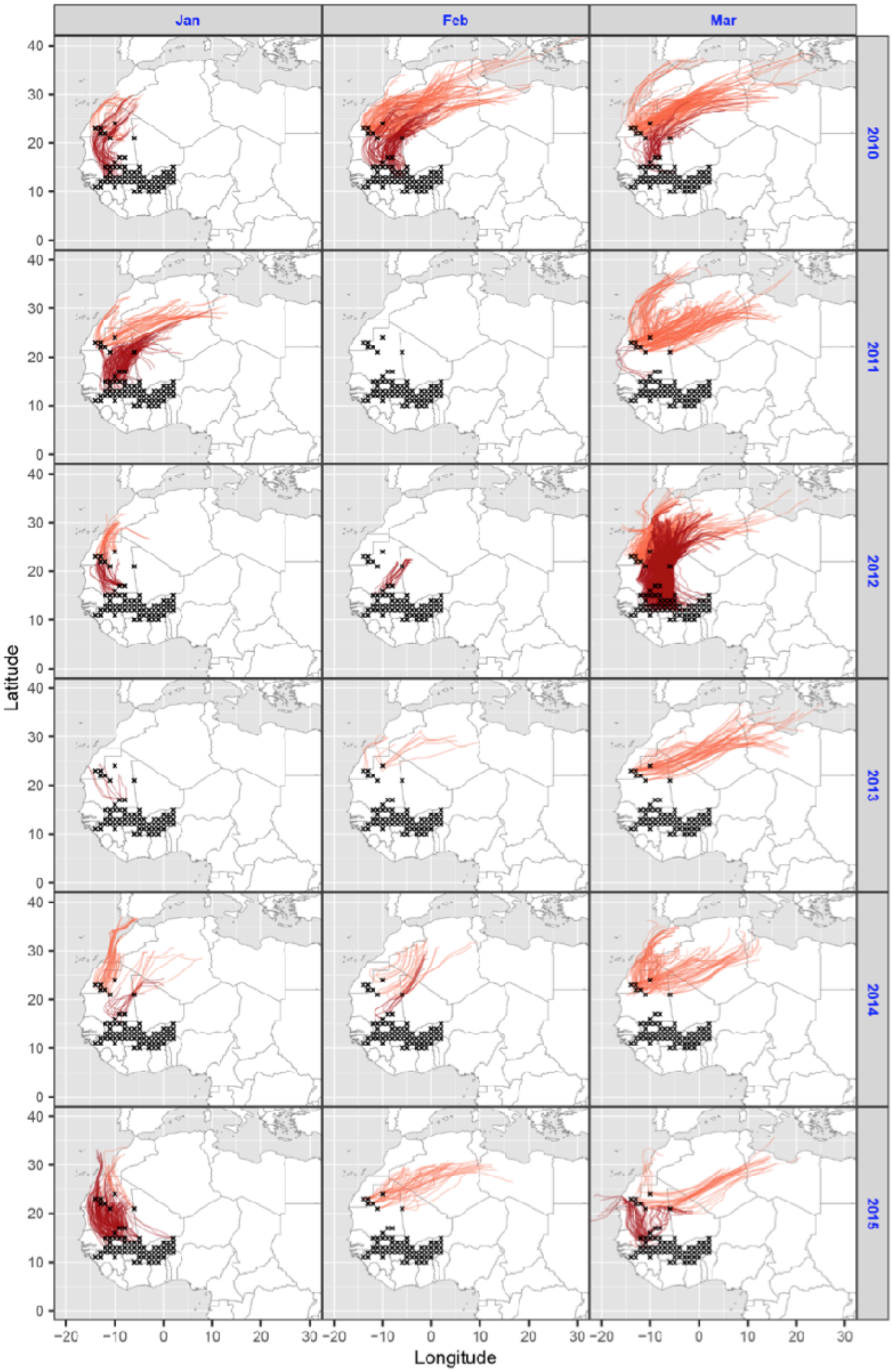
Jan
Feb
Mar
2010
2011
2012
2013
2014
2015
Latitude
Longitude
40
30
20
10
0
−20
−10
0
10
20
30

Fig. S5. Four-day trajectories from the west kernel. In these plots, all trajectories are shown, not just those that cross the Sahara or reach the northern sub-region.

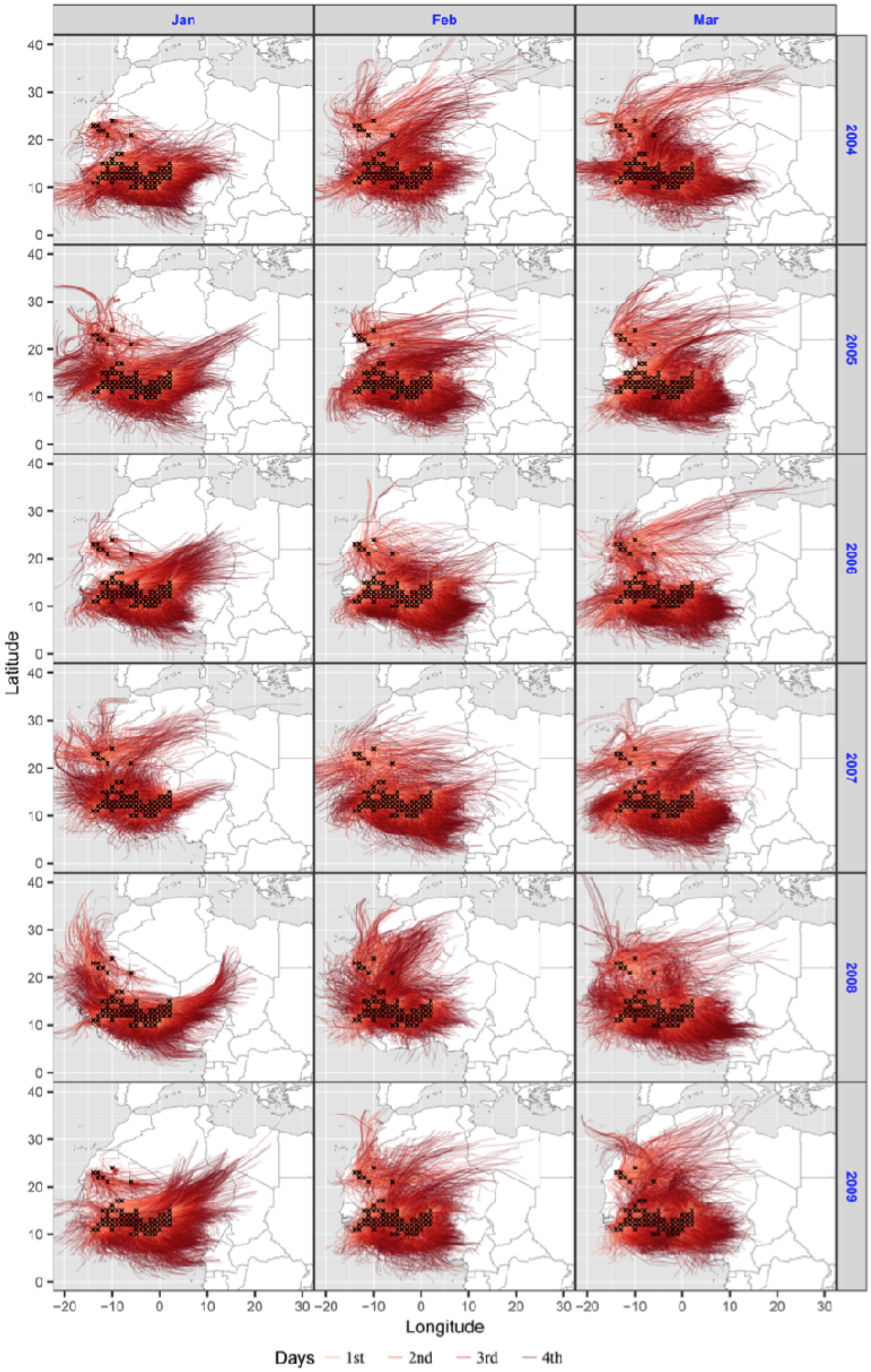

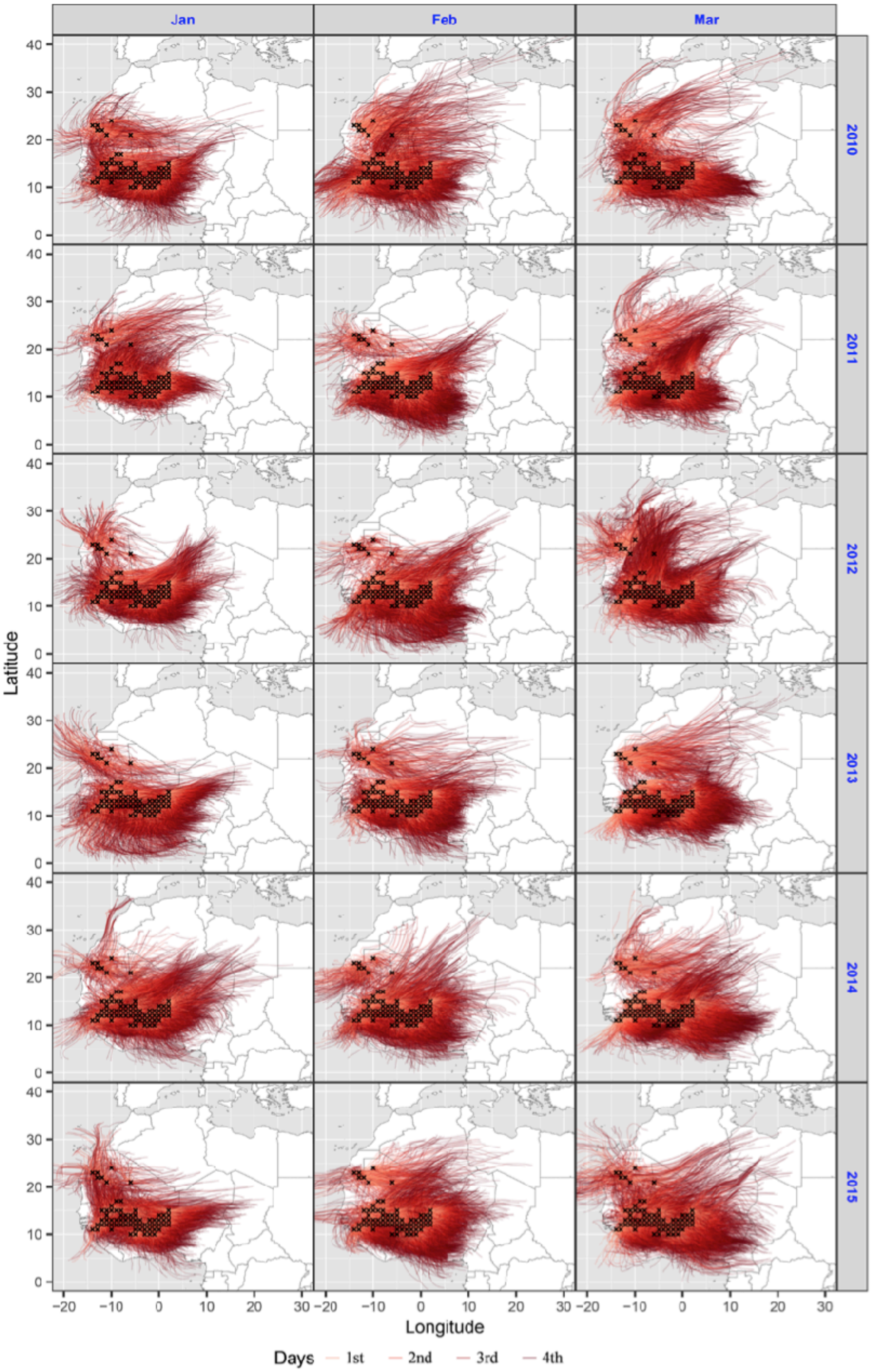

Jan
Feb
Mar
2010
2011
2012
2013
2014
2015
Latitude
Longitude
Days
1st
2nd
3rd
4th

Fig. S6. Four-day trajectories from the east kernel. In these plots, all trajectories are shown, not just those that cross the Sahara.

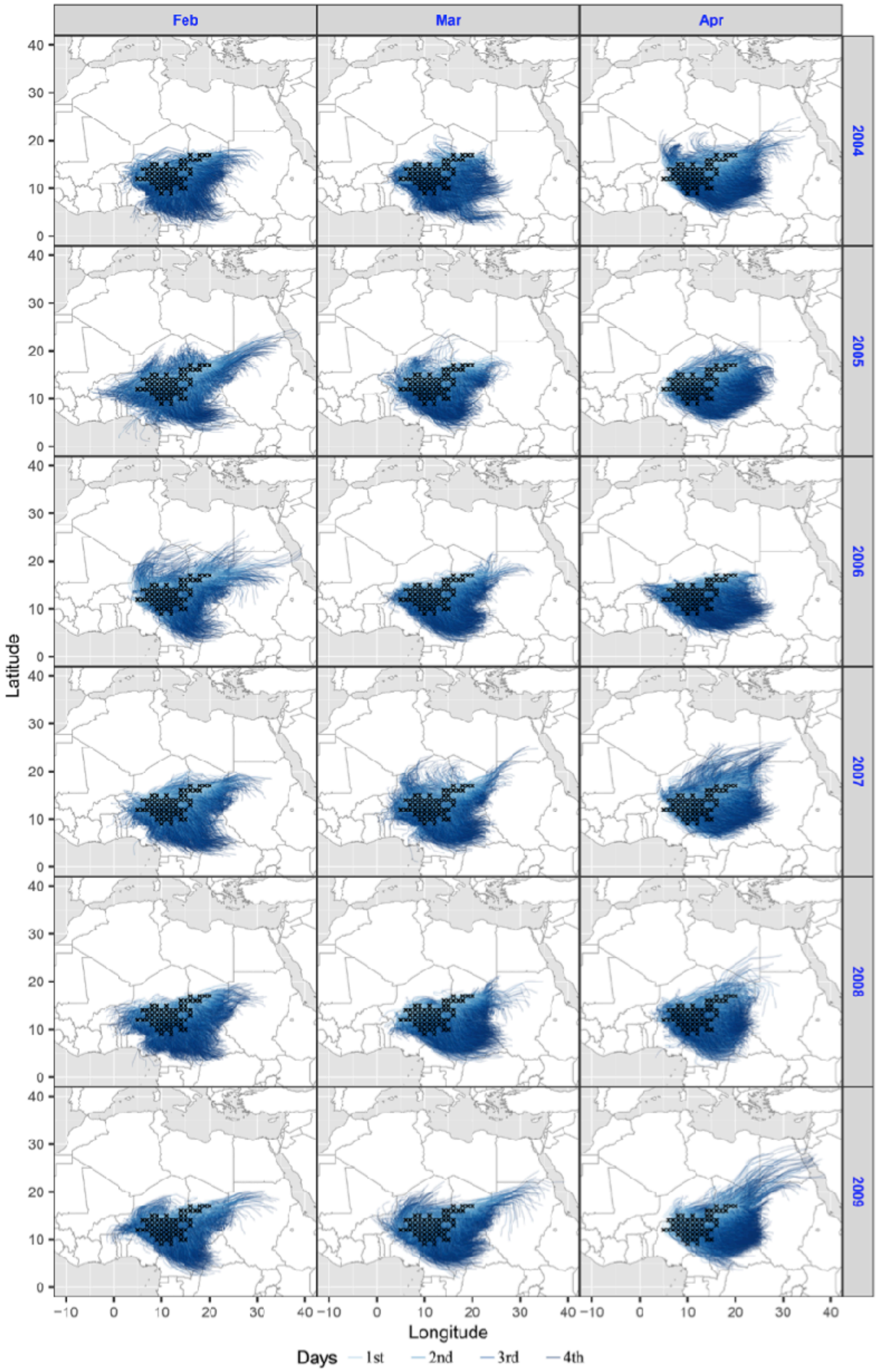

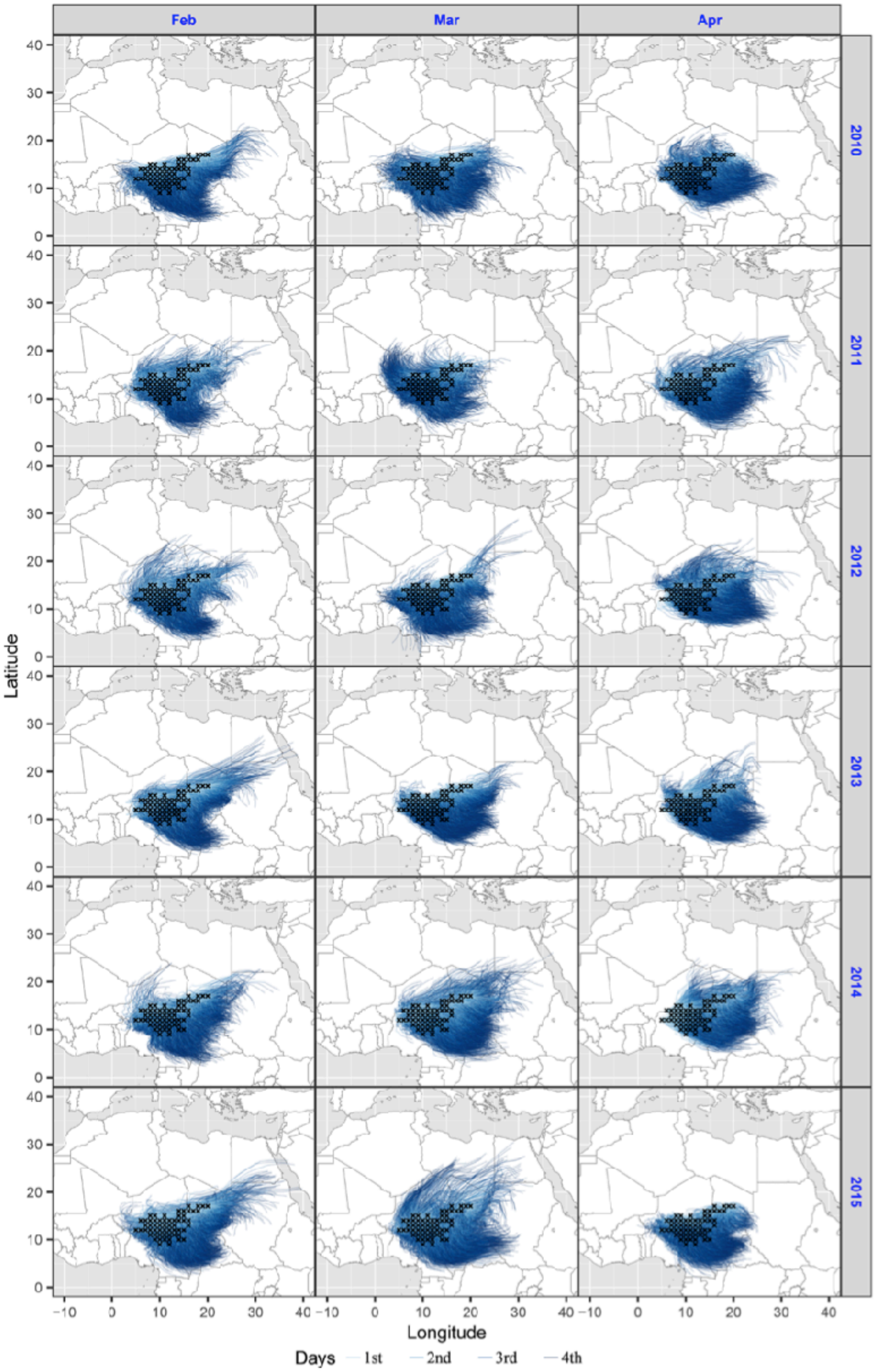
Feb
Mar
Apr
2010
2011
2012
2013
2014
2015
Latitude
Longitude
Days
1st
2nd
3rd
4th

Table S1. Description of statistical models used in the analysis.

Plus signs indicate additive effects of variables, whilst an asterisk (*) separating two variables indicates main effects of the two variables plus their interaction term.

Model	Variables included in model
**1**	**Full Model:**<br>$N_{Med.spring} = NDVI_{T.Forest} + NDVI_{Savannah} + NDVI_{Sahel} + NDVI_{Maghreb} + NDVI_{S.Iberia} + TL + DR + T + Y + \varepsilon$<br>**Minimum Model:**<br>$N_{Med.spring} = NDVI_{Savannah} + NDVI_{Maghreb} + TL + DR + T + Y + \varepsilon$
**2a**	**Full Model:**<br>$N_{Med.spring} = NDVI_{WEST.Kernel\_JAN} + NDVI_{WEST.Kernel\_FEB} + NDVI_{Maghreb} + TL + DR + T + Y + \varepsilon$<br>**Minimum Model:**<br>$N_{Med.spring} = NDVI_{WEST.Kernel\_JAN} + NDVI_{Maghreb} + TL + DR + T + Y + \varepsilon$
**2b**	**Full Model:**<br>$N_{Med.spring} = NDVI_{EAST.Kernel\_JAN} + NDVI_{EAST.Kernel\_FEB} + NDVI_{Maghreb} + TL + DR + T + Y + \varepsilon$<br>**Minimum Model:**<br>$N_{Med.spring} = NDVI_{EAST.Kernel\_FEB} + NDVI_{Maghreb} + TL + DR + T + Y + \varepsilon$
**3a**	$N_{Med.spring} = NDVI_{WEST.Kernel.JAN} * W_1 + TL + DR + T + Y + \varepsilon$
**3b**	$N_{Med.spring} = NDVI_{EAST.Kernel.FEB} * W_2 + TL + DR + T + Y + \varepsilon$
**4**	**Full Model:**<br>$N_{Med.summer} = D_{Med.spring} + NDVI_{NE.Spain} + T_{NE.Spain} + P_{NE.Spain} + NDVI_{Maghreb} + TL + DR + T + Y + \varepsilon$<br>**Minimum Model 4a:**<br>$N_{Med.summer} = D_{Med.spring} + T_{NE.Spain} + NDVI_{Maghreb} + TL + DR + T + Y + \varepsilon$<br>**Minimum Model 4b:**<br>$N_{Med.summer} = D_{Med.spring} + NDVI_{NE.Spain} + NDVI_{Maghreb} + TL + DR + T + Y + \varepsilon$<br>**Minimum Model 4c:**<br>$N_{Med.summer} = D_{Med.spring} + NDVI_{Maghreb} + TL + DR + T + Y + \varepsilon$
**5**	$N_{Eur.summer.early} = CI_{Med.summer} + NDVI_{Maghreb} + TL + DR + T + Y + \varepsilon$
**6**	**Full Model:**<br>$N_{Eur.summer.early} = CI_{Med.spring} + W_3 + W_4 + NDVI_{S.Iberia} + TL + DR + T + Y + \varepsilon$<br>**Minimum Model:**<br>$N_{Eur.summer.early} = CI_{Med.spring} + W_3 + TL + DR + T + Y + \varepsilon$
**7**	$N_{Eur.summer.late} = D_{Eur.summer.early} * T_{NW.Eur} + D_{Eur.summer.early} * P_{NW.Eur} + TL + DR + T + Y + \varepsilon$

Table S2. Statistical outputs for important variables in the models.

A colon (:) separating two variables indicates their interaction term; significant ($p < 0.05$) results, and those approaching significance ($p < 0.1$), of the best models are highlighted in bold text. Significance levels in all models:

^ p < 0.1   * p < 0.05   ** p < 0.01   *** p < 0.001   **** p < 0.0001

**Model**		**Principal variables**	**β Coefficient**	**S.E.**	**z**	**P**
**1** ($N_{Med.spring}$)	Full Model:	$NDVI_{T.Forest}$	-0.135	0.318	-0.424	0.672
		$NDVI_{Savannah}$	0.908	0.507	1.793	0.073 ^
	n = 1076	$NDVI_{Sahel}$	-0.649	0.528	-1.231	0.218
		$NDVI_{Maghreb}$	0.385	0.299	1.289	0.197
		$NDVI_{S.Iberia}$	0.421	0.326	1.289	0.198
	Minimum Model:	$NDVI_{Savannah}$	0.444	0.250	1.776	**0.076 ^**
		$NDVI_{Maghreb}$	0.512	0.256	1.999	**0.046 ***
**2a** ($N_{Med.spring}$)	Full Model:	$NDVI_{WEST.Kernel\_JAN}$	0.567	0.288	1.968	0.049 *
		$NDVI_{WEST.Kernel\_FEB}$	-0.073	0.293	-0.251	0.801
	n = 1076	$NDVI_{Maghreb}$	0.481	0.256	1.881	0.060
	Minimum Model:	$NDVI_{WEST.Kernel\_JAN}$	0.528	0.244	2.161	**0.031 ***
		$NDVI_{Maghreb}$	0.496	0.249	1.992	**0.046 ***
**2b** ($N_{Med.spring}$)	Full Model:	$NDVI_{EAST.Kernel\_JAN}$	-0.108	0.273	-0.395	0.693
		$NDVI_{EAST.Kernel\_FEB}$	0.763	0.291	2.620	0.009 **
	n = 1076	$NDVI_{Maghreb}$	0.556	0.227	2.451	0.014 *
	Minimum Model:	$NDVI_{EAST.Kernel\_FEB}$	0.690	0.227	3.043	**0.002 ****
		$NDVI_{Maghreb}$	0.561	0.227	2.469	**0.014 ***
**3a** ($N_{Med.spring}$)	Full Model:	W_1	0.314	0.287	1.093	0.274
	n = 1076	$NDVI_{WEST.Kernel.JAN}$: W_1	0.588	0.288	2.040	**0.041** *
**3b** ($N_{Med.spring}$)	Full Model:	W_2	0.270	0.292	0.927	0.354
	n = 1076	$NDVI_{EAST.Kernel.JAN}$: W_2	-0.342	0.391	-0.877	0.381

**4** ($N_{Med.summer}$)	Full Model:	$D_{Med.spring}$	0.035	0.008	4.469	**<0.0001** ****
	n = 1070	$NDVI_{NE.Spain}$	-0.449	0.328	-1.366	0.172
		$T_{NE.Spain}$	-0.408	0.311	-1.308	0.191
		$P_{NE.Spain}$	-0.039	0.321	-0.121	0.904
		$NDVI_{Maghreb}$	0.719	0.252	2.852	**0.004** **
	Minimum Model 4a:	$D_{Med.spring}$	0.035	0.008	4.478	**<0.0001** ****
		$T_{NE.Spain}$	-0.566	0.258	-2.195	**0.028** *
		$NDVI_{Maghreb}$	0.572	0.240	2.389	**0.017** *
	Minimum Model 4b:	$D_{Med.spring}$	0.035	0.008	4.457	**<0.0001** ****
		$NDVI_{NE.Spain}$	-0.635	0.290	-2.190	**0.028** *
		$NDVI_{Maghreb}$	0.784	0.259	3.028	**0.002** **
	Minimum Model 4c:	$D_{Med.spring}$	0.035	0.008	4.464	**<0.0001** ****
		$NDVI_{Maghreb}$	0.567	0.264	2.145	**0.032** *
**5** ($N_{Eur.summer.early}$)	Full Model: n = 21,554	$CI_{Med.summer}$	0.830	0.165	5.024	**<0.0001** ****
		$NDVI_{Maghreb}$	0.178	0.230	0.772	0.440
**6** ($N_{Eur.summer.early}$)	Full Model:	$CI_{Med.spring}$	1.358	0.181	7.495	**<0.0001** ****
		W_3	0.429	0.233	1.847	0.065 ^
	n = 21,554	W_4	0.064	0.221	0.288	0.773
		$NDVI_{S.Iberia}$	0.189	0.199	0.948	0.343
	Minimum Model:	$CI_{Med.spring}$	1.423	0.175	8.120	**<0.0001** ****
		W_3	0.403	0.175	2.301	**0.021** *
**7** ($N_{Eur.summer.late}$)	Full Model:	$D_{Eur.summer.early}$	0.047	0.002	24.48	**<0.0001** ****
	n = 21,554	$T_{NW.Eur}$	-0.031	0.324	-0.095	0.924
		$P_{NW.Eur}$	-0.527	0.330	-1.595	0.111
		$D_{Eur.summer.early}$: $T_{NW.Eur}$	0.036	0.002	21.74	**<0.0001** ****
		$D_{Eur.summer.early}$: $P_{NW.Eur}$	0.015	0.002	6.369	**<0.0001** ****

Abbreviations for Tables S1 & S2

Note: "PL" = painted lady butterfly. "WSS" = West Sudanian Savannah

$N_{Med.spring}$ – PL count in NE Spain in the spring period (**Mar–May**)
$N_{Med.summer}$ – PL count in NE Spain in the summer period (**June–July**)
$N_{Eur.summer.early}$ – PL count in NW Europe in early summer (**15 May–15 July**)
$N_{Eur.summer.late}$ – PL count in NW Europe in late summer (**16 July–30 Sept**)
$D_{Med.spring}$ – Density (PL counts/km) in NE Spain in spring (**Mar–May**)
$D_{Eur.summer.early}$ – Density (PL counts/km) in NW Europe in early summer (**15 May–15 July**)
$CI_{Med.summer}$ – Annual collated PL summer abundance index in NE Spain

$NDVI_{T.Forest}$ – NDVI in the tropical forest region in **Jan–Feb**
$NDVI_{Sahel}$ – NDVI in the Western Sahel region in **Jan–Feb**
$NDVI_{Savannah}$ – NDVI in the West Sudanian Savannah (WSS) region in **Jan–Feb**
$NDVI_{WEST.Kernel.JAN}$ – NDVI in the **West Kernel** of the WSS in **Jan**
$NDVI_{WEST.Kernel.FEB}$ – NDVI in the **West Kernel** of the WSS in **Feb**
$NDVI_{EAST.Kernel.JAN}$ – NDVI in the **East Kernel** of the WSS in **Jan**

$NDVI_{EAST.Kernel.FEB}$ – NDVI in the **East Kernel** of the WSS in **Feb**
$NDVI_{Maghreb}$ – NDVI in the Maghreb in **Mar–Apr**
$NDVI_{S.Iberia}$ – NDVI in Southern Iberia in **Apr–May**
$NDVI_{NE.Spain}$ – NDVI in NE Spain in **Mar–May**

W1 – Southerly wind speed (v) at 1500 m from West Kernel to North Africa (**15 Feb–15 Mar**)
W2 – Southerly wind speed (v) at 1500 m from East Kernel to North Africa (**15 Feb–15 Mar**)
W3 – Southerly wind speed (v) at 1500 m from NE Spain to NW Europe in **June**
W4 – Westerly wind speed (u) at 1500 m from NE Spain to NW Europe in **June**

$T_{NE.Spain}$ – Mean air temperature in °C in spring (**Mar–May**) in NE Spain
$T_{NW.Eur}$ – Mean air temperature in °C in summer (**June–July**) in NW Europe
$P_{NE.Spain}$ – Total precipitation (mm) in spring (**Mar–May**) in NE Spain
$P_{NW.Eur}$ – Total precipitation (mm) in summer (**June–July**) in NW Europe

TL – Transect length in meters
DR – Number of days transect was recorded
Year – Fixed effect for year when PLs were recorded
T – random intercept for transect ID
Y – random intercept for year
ε – Poisson error term

Table S3. Number of "successful" trajectories arriving in the Maghreb or the northern section of the west kernel.

Year	Month	No. arriving in Maghreb from entire 'west kernel'	No. arriving in Maghreb from northern section of 'west kernel'	No. arriving in Maghreb from southern section of 'west kernel'	No. arriving in northern section of 'west kernel' from southern section
2004	January	2	2	0	0
2004	February	155	149	6	23
2004	March	132	116	16	169
2005	January	20	20	0	69
2005	February	23	23	0	28
2005	March	99	99	0	0
2006	January	6	6	0	0
2006	February	22	22	0	4
2006	March	80	80	0	40
2007	January	106	101	5	102
2007	February	30	30	0	5
2007	March	30	30	0	0
2008	January	2	2	0	76
2008	February	344	260	84	282
2008	March	71	71	0	49
2009	January	5	2	3	0
2009	February	126	82	44	73

2009	March	183	181	2	61
2010	January	29	22	7	45
2010	February	138	133	5	102
2010	March	155	149	6	53
2011	January	60	58	2	129
2011	February	0	0	0	0
2011	March	187	184	3	4
2012	January	37	37	0	42
2012	February	0	0	0	0
2012	March	356	203	153	556
2013	January	0	0	0	0
2013	February	10	10	0	0
2013	March	61	61	0	0
2014	January	48	48	0	14
2014	February	59	21	38	9
2014	March	110	110	0	0
2015	January	18	15	3	163
2015	February	41	41	0	0
2015	March	48	39	9	53
	**Total**	**2792**	**2406**	**386**	**2152**
	**Mean**	**77.6**	**66.8**	**10.7**	**59.7**
